# Promoter activity dynamics in the lag phase of *Escherichia coli*

**DOI:** 10.1186/1752-0509-7-136

**Published:** 2013-12-30

**Authors:** Daniel Madar, Erez Dekel, Anat Bren, Anat Zimmer, Ziv Porat, Uri Alon

**Affiliations:** 1 Department of Molecular Cell Biology, Weizmann Institute of Science, Rehovot 76100, Israel; 2 Biological Services Unit, Weizmann Institute of Science, Rehovot 76100, Israel

**Keywords:** E. coli, Lag phase, Resource allocation, Optimal control theory, Bang-bang control, Pontryagin maximum principle, Transcriptional program, Stringent response

## Abstract

**Background:**

Lag phase is a period of time with no growth that occurs when stationary phase bacteria are transferred to a fresh medium. Bacteria in lag phase seem inert: their biomass does not increase. The low number of cells and low metabolic activity make it difficult to study this phase. As a consequence, it has not been studied as thoroughly as other bacterial growth phases. However, lag phase has important implications for bacterial infections and food safety. We asked which, if any, genes are expressed in the lag phase of *Escherichia coli*, and what is their dynamic expression pattern.

**Results:**

We developed an assay based on imaging flow cytometry of fluorescent reporter cells that overcomes the challenges inherent in studying lag phase. We distinguish between lag1 phase- in which there is no biomass growth, and lag2 phase- in which there is biomass growth but no cell division. We find that in lag1 phase, most promoters are not active, except for the enzymes that utilize the specific carbon source in the medium. These genes show promoter activities that increase exponentially with time, despite the fact that the cells do not measurably increase in size. An oxidative stress promoter, *katG*, is also active. When cells enter lag2 and begin to grow in size, they switch to a full growth program of promoter activity including ribosomal and metabolic genes.

**Conclusions:**

The observed exponential increase in enzymes for the specific carbon source followed by an abrupt switch to production of general growth genes is a solution of an optimal control model, known as bang-bang control. The present approach contributes to the understanding of lag phase, the least studied of bacterial growth phases.

## Background

When bacteria are inoculated into fresh medium, they often show a period without growth known as the lag phase [1-3]. Lag phase is interesting as a fundamental biological process in which bacterial physiology adapts to a new environment. Lag phase is also of interest in fields such as food safety- in which lag phase is one factor in determining food shelf life [4-6]. In medicine, lag phase plays a role when bacteria move into the blood stream or other locations where rapid growth can occur. In both food safety and medicine, the longer the lag phase the longer body defenses have time to fight pathogens, and the longer the natural gut flora have to out-compete the pathogens. In some cases, longer lag phase is associated with increased invasiveness of bacterial cells [7,8], or with antibiotic tolerance [9].

**Figure 1:**
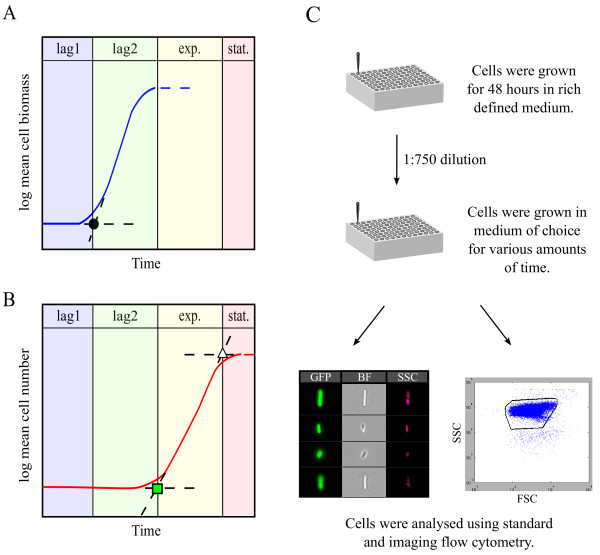
**Definition of lag1 and lag2 phases, and the experimental system. (A)** A schematic representation of mean cell biomass over time, with separation into phases. In lag1, biomass does not measurably increase. The end of lag1 is operationally defined using the biomass (or cell length) curve, at the intersection between the constant biomass line at the beginning of the curve, with the extrapolated exponential increase in biomass in lag2 (black circle). In lag2, biomass increases. The end of lag2 is defined by the first division events. At this point biomass plateaus (or peaks and then declines), as cells reach a biomass characteristic of exponential growth. See Additional file 1: Table S1, and Figure S1. **(B)** A schematic representation of mean cell number over time, with separation into phases. The end of lag2 is operationally defined by the intersection between the constant cell number line at the beginning of the curve, with the extrapolated exponential increase in cell number (green rectangle). The end of exponential phase is operationally defined by the intersection between the increasing cell number line, with the constant cell number line at the end of the curve (white triangle). See Additional file 1: Table S1, and Figure S1. **(C)** Schematic description of the experiment: cells were grown for 48 hours in rich defined medium, inoculated into poor or rich defined medium, grown for various amounts of time in a robotic system, and analyzed by standard flow cytometry and by imaging flow cytometry.

There are several definitions of lag phase [2-4,10-18] -See Additional file 1: Table S1 for details. For clarity, we divide lag phase into two sub-phases: lag1 is the period between inoculation and until biomass begins to grow, and lag2 is the period between the end of lag1 and the time that cell numbers begin to increase (Figure 1A, B and Additional file 1: Figure S1). Lag1 has also been called the adaptation phase [13], a term we do not use because of the multiple meanings of adaptation in sensing and evolution. Note that lag1 is not adaptation by mutation, but rather acclimatization of the cell physiology to new conditions. The name 'lag2' is contradictory in the sense that it is a phase of growth, namely the first cell generation. We however chose this name to describe the second part of the lag phase because cells do not grow in number, which is why previous works considered this phase to be part of lag phase.

Operationally, the length of lag1 phase is determined in a logarithmic plot of biomass or a related quantity (cell length, optical density, dry weight) by extrapolating the exponential growth of biomass and finding its intersection with the initial biomass (Figure 1A). The end of lag2 can be similarly defined from a logarithmic graph of cell numbers (Figure 1B).

When cells are grown overnight in a rich medium and then transferred to a poor medium, both lag1 and lag2 phases may occur. Mathematical models of lag phase include Baranyi’s model [19] in which biomass production relies on accumulation of bottleneck proteins in lag phase [18,19], and models which focus on cell-cell variation in lag phase duration [4,20]. Each individual cell has lag1 and lag2 phases, but the duration of these phases varies stochastically between individual cells, as is evident in the measurements of Métris et al. [21] (Additional file 1: Figure S1). One possible origin of these cell-cell variations is stochastic variation in the levels of enzymes present in the cell [22-25].

Individual cell measurements also show that the cell cycle accelerates during the first few divisions [20,21,26], and thus it takes several generations to reach the maximal exponential growth rate. Thus, lag2 as defined here is only the first generation in a prolonged acceleration phase [3] (Additional file 1: Figure S1).

Studies of gene expression in bacterial lag phase are relatively few [27-29]. Several studies have focused on particular genes, such as the transcription regulator *fis*[30] and ribosomal genes [31-33]. A recent study explored the genome-wide expression patterns of *Salmonella Typhimurium* upon transition from stationary phase in rich medium (48 hours growth in LB) to fresh LB medium [34]. In this case, bacteria seem to make biomass almost immediately (lag2), making it difficult to resolve lag1 phase. The expression program in lag2 was similar to expression in exponential growth, with some differences in genes related to metal ion accumulation, phosphate accumulation and oxidative stress. Thus, current knowledge is greatest for lag2, the phase in which biomass grows, suggesting that it is similar in many ways to exponential phase.

In contrast to lag2, there is little knowledge about lag1 phase, the phase in which biomass does not grow. This lack of knowledge is due in part to the technical difficulty of studying lag1, and in part to the fact that commonly used protocols go from rich medium to rich medium so that lag1 is very short. It is therefore not clear whether the expression program in lag1 is fundamentally different from the expression in lag2 and exponential growth. For example, one may imagine several possibilities: either no expression in lag1 of any gene, expression that is directed only towards selected genes, or expression of the same genes as in exponential phase but at low intensity.

Here, we address the nature of lag phase by developing an assay for measuring cell size, cell number and promoter activity at high accuracy in individual bacterial cells in lag1 and lag2 phases. This assay allows us to overcome the problem of low signal to background that occurs in culture-based assays of lag phase that rely on fluorimeters for optical density (OD) and fluorescence measurements. We found that the expression program in lag1 phase is very different from that in lag2 and exponential phases. Most promoters are not measurably active in lag1 phase. The promoters of the utilization operons for the specific carbon source in the medium are, however, highly active in lag1 phase showing an exponential increase with time, despite the fact that biomass does not measurably increase. In lag2 and exponential phase, utilization genes, ribosomal genes and a wide range of growth genes are expressed together. This suggests a ‘bang-bang’ control strategy, known from optimal control theory in engineering: first concentrate resources on generating utilization genes for the specific carbon source, so that carbon can flow into energy production and building blocks (such as amino acids), and only then make ribosomes to produce biomass. The seemingly inert lag1 phase thus shows selective and strong expression of specific genes.

## Results

### Flow cytometry assay on fluorescent reporter cells allows differentiation between bacterial lag1 and lag2 growth phases

To measure promoter activity, we used strains from the *E. coli* reporter strain library previously developed in our lab [35-37]. Each strain bears a low-copy plasmid with fast folding GFP under the control of a full length copy of the promoter of interest.

A technical challenge for studying lag1 is low cell density: one cannot use the reporter strains to measure promoter activity during lag1 phase in a culture using a multi-well fluorimeter, because cell density and fluorescence is below the background detection limit [4,38]. One can use a high inoculum level (low dilution of stationary phase starters) to bypass the OD detection threshold, but this can affect lag phase duration and behavior due to the relatively high concentration of stationary phase stimulatory and inhibitory molecules [39-41], and due to quorum sensing [42-44]. Thus, we developed a flow cytometry assay to measure fluorescence and cell size from individual cells grown in batch culture (Figure 1C).

To prepare the cells for flow cytometry at multiple time points after inoculation, we used a robotic liquid handling system to transfer cells from stationary phase culture to 96- deep well plates in a time-delayed manner. Wells were inoculated at varying temporal intervals, ranging from 5 to 180 minutes. At the end of this procedure, the multi-well plate had cells grown in fresh medium for different amounts of time post inoculation, ranging from zero to 15 hours. Cells were kept on ice and measured in flow cytometers, so that samples represented a post-inoculation time course.

We used flow cytometry to measure cell number and individual cells' fluorescence. By viewing individual cells, flow cytometry overcomes the signal to noise problems of low density batch cultures. We measured total cell density by the number of cytometry counts at a given time point. This measurement of cell density agreed very well with colony-forming-unit measurements on agar plates (Additional file 1: Figure S4).

We also measured cells in an imaging flow cytometer, which provides images of individual cells. These images allowed a direct measurement of cell length. We used cell length to operationally define the end of lag1 phase, at the intersection between the initial cell length line at the beginning of the curve, with the extrapolated exponential increase in cell length in lag2 (Figure 1A, black circle). The end of lag2 is operationally defined using the cell number curve: it is the intersection of the cells' exponential growth line with the line of initial cell number (Figure 1B, green rectangle). The end of exponential phase is similarly defined as the intersection between the exponential growth line and the line of final cell density (Figure 1B, white triangle).

**Figure 2:**
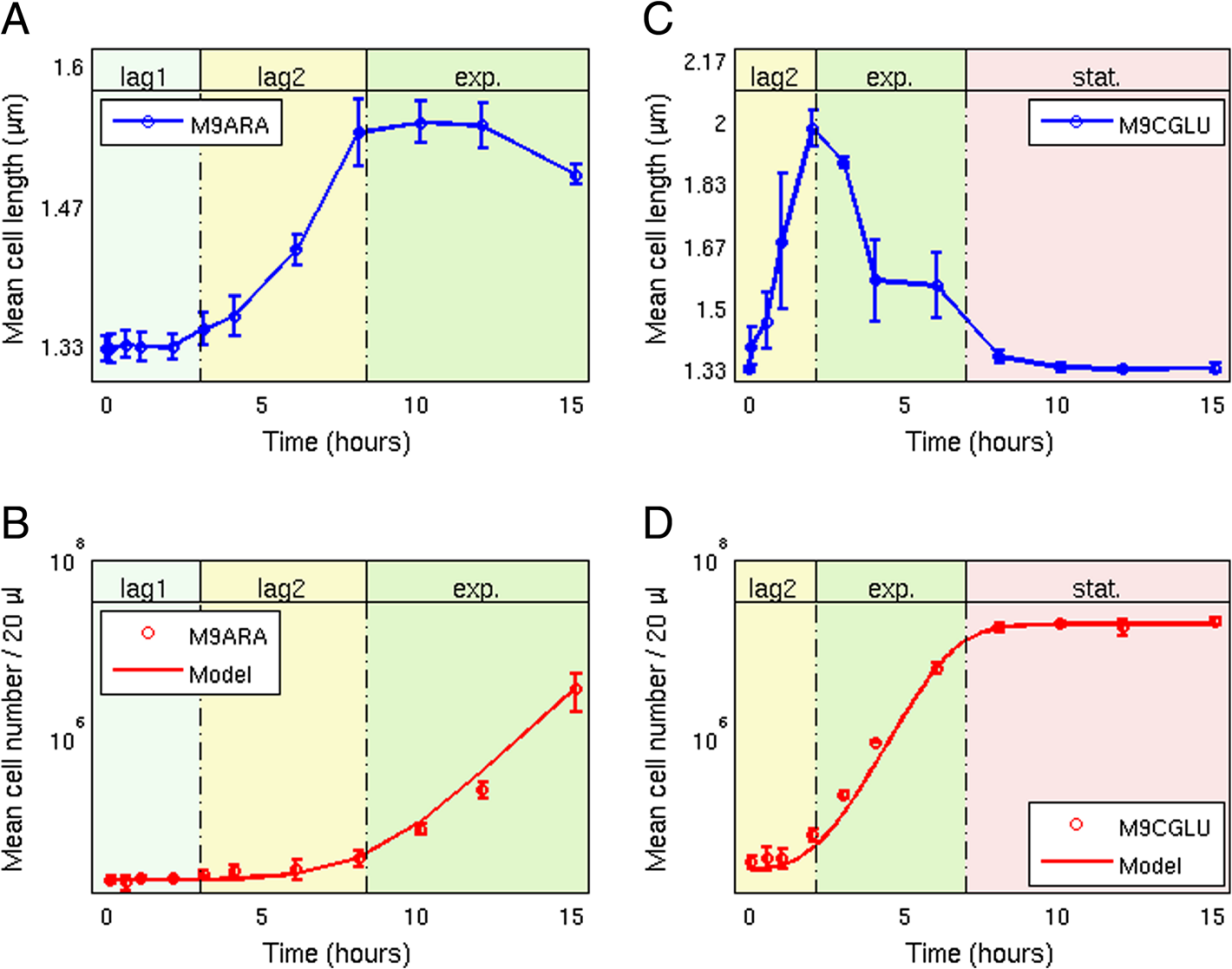
**Cell length and cell number measurements allow growth curve segmentation into lag1 and lag2 phases. (A)** Mean bacterial length in minimal arabinose medium (M9ARA). 1 μm = 3 pixels. The blue line is a guide to the eye. **(B)** Mean cell numbers (in 20 μl sample) in M9ARA medium. The red line is a fit to the optimal control model. **(C)** Mean bacterial length in minimal glucose + amino-acid medium (M9CGLU). 1 μm = 3 pixels. The blue line is a guide to the eye. **(D)** Mean cell number (in 20 μl sample) in M9CGLU medium. The red line is a fit to the model. Error bars indicate standard error.

We studied cells transferred from a stationary phase culture in defined M9 medium with glucose (0.2%) and casamino acids (M9CGLU), into fresh M9 medium with no casamino acids in which a given sugar (arabinose) is the sole carbon source at saturating (0.2%) concentrations (M9ARA). As a control, we also studied cells inoculated into fresh M9CGLU. We found that for M9ARA, a significant lag1 phase was seen lasting for 3 ± 1 hours (Figure 2A). After the lag1 period, cells began to grow in length and entered lag2. The duration of lag2 was 5.4 ± 1.2 hours. Thus lag1 + lag2 lasted for 8.4 ± 1.2 hours. When cells entered exponential phase, the rise in cell numbers coincided with the plateauing of mean cell length (Figure 2B and Figure 2A respectively). This is because cell divisions prevent a further rise in mean cell length. The exponential growth rate was 0.76 ± 0.07 hour^-1^, corresponding to a generation time of 55 ± 5 minutes.

In contrast, transfer to the richer medium with glucose and amino acids (M9CGLU) showed immediate growth in cell size, and thus cells entered lag2 without a measurable delay (Figure 2C). Here, the rise in cell number is also coupled to the peak of mean cell size (Figure 2D and Figure 2C respectively). The duration of lag2 was 2.2 ± 0.5 hours, the exponential growth rate is 1.4 ± 0.4 hour^-1^, and the exponential generation time was 29 ± 8 minutes. When cells entered stationary phase, their mean length decreased back to its pre-inoculation level (Figure 2C).

### In lag1 phase, genes for arabinose utilization are expressed exponentially with time despite no growth in biomass

**Figure 3:**
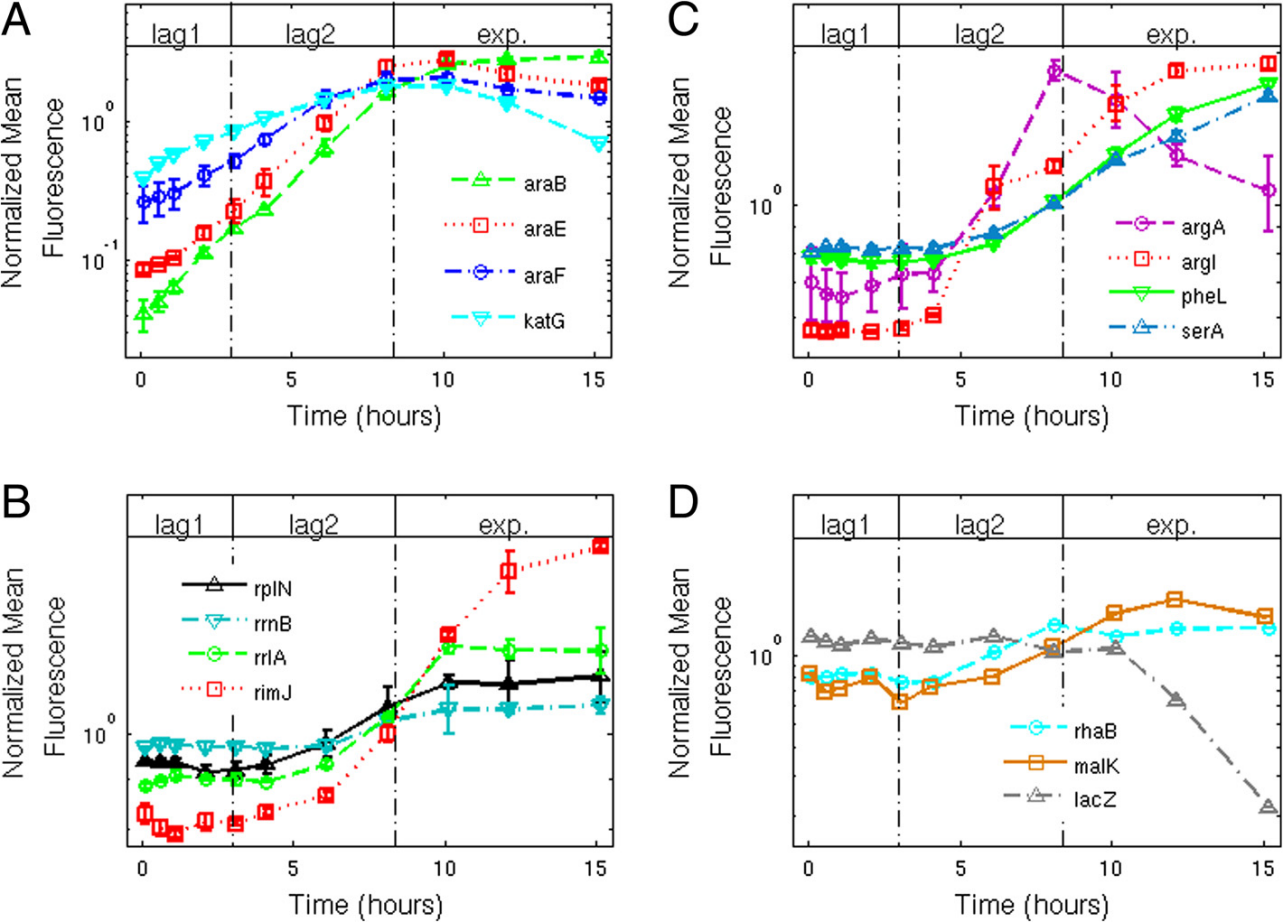
**On arabinose minimal medium, the arabinose promoters are selectively activated during lag1.** Mean GFP fluorescence of the cells in minimal arabinose medium (M9ARA), for different reporter strains. Growth phases are indicated. Data for each reporter was normalized by its mean value.. Lines are guides to the eye. Error bars indicate standard error. **(A)** Normalized mean fluorescence of the arabinose system reporters, and of *katG*. See Additional file 1: Figure S3. **(B)** Normalized mean fluorescence of ribosomal genes reporters. **(C)** Normalized mean fluorescence of amino-acid genes reporters. **(D)** Normalized mean fluorescence of the rhamnose, maltose and lactose system reporters.

We measured the fluorescence of the reporter cells for genes related to arabinose utilization in fresh M9ARA medium. We found that the fluorescence of the arabinose utilization genes rises exponentially with time during lag1 and lag2 phases. The reporters for the catabolic operon *araBAD*, and the transporters *araE* and *araFGH*, show an exponential rise in fluorescence despite no measurable increase in biomass in lag1. *araBAD* expression rose by a factor of about 60 over the eight hours of lag1 + lag2, (Figure 3A), corresponding to an exponential rate of 0.45 ± 0.02 hour^-1^ and a doubling time of 92 ± 4 minutes. Similarly, *araE* showed an exponential increase of about 60 fold, at rate 0.44 ± 0.03 hour^-1^. This doubling time is nearly twice the cell doubling time of 55 ± 5 minutes in exponential phase in the same medium. As cells leave lag2 phase and begin to divide, these reporters reach a steady-state of fluorescence levels, because cell divisions dilute the GFP produced by these promoters.

One possible explanation for the exponential rise in reporter fluorescence during lag1 is an auto-catalytic process in which cells devote their gene expression resources to utilizing the sugar; the more transporters available, the more the sugar can be utilized giving rise to more expression and so on.

Individual cell data from the flow cytometry experiment indicates that the expression of the arabinose system promoter increases with time for all cells, and is not an all-or-none phenomenon in which some cells express the operons and others do not (Additional file 1: Figure S2).

### In lag1 phase, growth genes are not measurably expressed

We also tested ribosomal promoter activity in M9ARA medium, using relevant reporter strains (*rplN*, *rrnB, rrlA, rimJ*). We found that ribosomal reporters did not show an appreciable rise during lag1 phase in arabinose (Figure 3B). When cells began to grow in size (lag2 phase), the ribosomal promoters were activated (Figure 3B). This lag2 increase appears mild in the case of *rrnB* due to its relatively high initial level. The promoters began to approach a steady-state activity levels in exponential phase, when their mean GFP production rates are balanced by the dilution rate due to cell division.

We also tested amino-acid biosynthesis promoters for arginine, phenylalanine and serine (*argA, argI, pheL* and *serA*). We found that these promoters were not measurably active during lag1 on arabinose (Figure 3C), even though the medium did not contain amino acids. These promoters became active only in lag2, rising at different rates, approaching a steady-state in exponential phase.

We tested catabolic genes for other sugars, rhamnose, maltose and lactose (*rhaB, malK* and *lacZ*). We found that these promoters were also not measurably active in lag1 or thereafter (Figure 3D). Thus, cells seemed to selectively express the utilization genes for the nutrient in the medium (arabinose) in lag1, and not utilization genes for other sugars.

To extend our survey of promoter activity in lag1 phase, we performed a screen using 140 additional promoters, with diverse biological functions, in M9ARA medium. The promoters controlled genes involved in translation, transcription, global regulators, amino acid biosynthesis, oxidative stress, phosphate and metal transport and metabolism, cold shock, heat shock and global stress (see Additional files 1, 2 and 3). To allow a large screen, we used fewer time-points: three in lag1 and one in lag2 phase. No growth related promoters (ribosomal, transcriptional, translational, amino-acid biosynthesis, etc.) showed a measurable promoter activity. Genes with a significant increase in lag1 were re-tested at higher temporal resolution. We find that only one promoter reproducibly showed more than a 3-fold increase during lag1, the oxidative stress promoter *katG* (Figure 3A). However, it does not rise as rapidly as the arabinose promoters. We also found a minor increase in some of the iron metabolism and transporter promoters (see Additional files 2 and 3).

**Figure 4:**
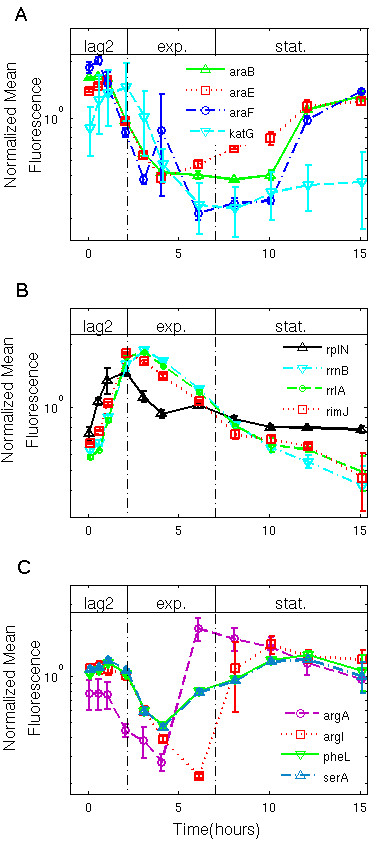
**Cells enter lag2 immediately on glucose minimal medium, and express ribosomal promoters.** Normalized mean GFP fluorescence of the cells over time, for different reporter strains in minimal glucose + amino-acid medium (M9CGLU). Growth phases are indicated. Data for each reporter were normalized by its mean value. Lines are guides to the eye, except for the *katG* reporter, in which the data was smoothed by a Gaussian lowpass filter. Error bars **(A)** Normalized mean fluorescence of the arabinose system reporters, and *katG.***(B)** Normalized mean fluorescence of ribosomal reporters. **(C)** Normalized mean fluorescence of amino-acid genes reporters.

When cells were inoculated into glucose and amino acid medium (M9CGLU medium), instead of arabinose, they skipped lag1 and entered lag2 immediately. We found that the arabinose utilization promoters were not active during lag2 or exponential phases, and slightly rose at stationary phase when glucose is depleted and cAMP rises (Figure 4A). Ribosomal promoters were active immediately after inoculation, during lag2 and exp. phase (Figure 4B). The amino-acid promoters were mostly activated towards the end of the exp. phase, when the medium ran out of amino-acids (Figure 4C).

### Results are consistent with optimal control principle of ‘bang-bang’ control

Taken together, these results suggest that in lag1 on arabinose, cells mostly focus their promoter activity on the arabinose utilization genes. They do not activate the vast majority of promoters, including ribosomal genes, amino acid biosynthesis genes or utilization genes for other sugars.

**Figure 5:**
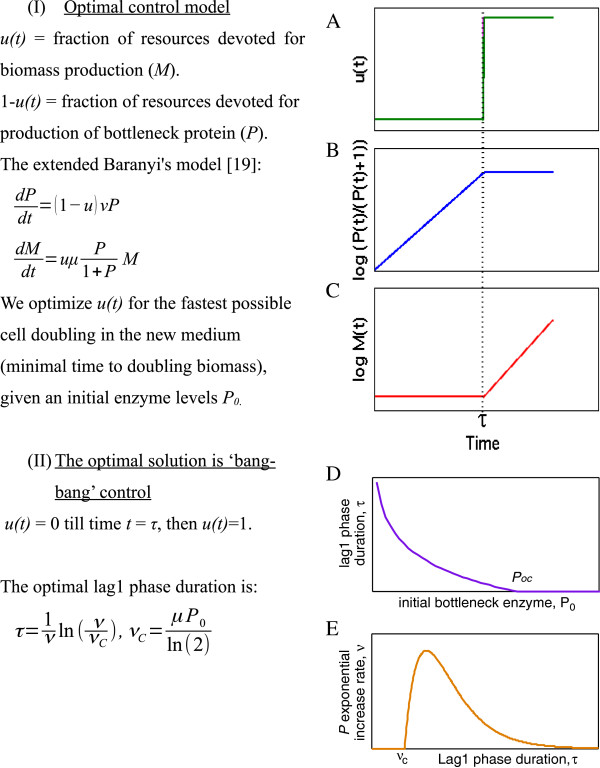
**An optimal control model suggests a sudden shift from making bottleneck proteins to full biomass production. (A)** The model seeks the optimal allocation function *u(t)* between making bottleneck protein *P* and making biomass *M*, in order to minimize the time to double the biomass.. The function *u(t)* is the fraction of resources devoted to making *M*, and *1-u(t)* the fraction devoted to making *P.* The rate of production of *P* is parameterized by a maximal rate *ν*. The maximal rate of producing *M* is the exponential growth rate *μ*. A logistic term is omitted for simplicity, and including it does not change the qualitative result. The optimal solution for *u(t)*, obtained in the Methods section, is a sharp switch from *u = 0* to a value of *u = 1*, at time *τ*. Lag1 is the time up to time τ, in which the cells make only *P*. In lag2 and exponential phases, the period of time after τ, the cells make both bottleneck protein *P* and biomass *M*. **(B)** The level of the bottleneck protein *P*. See Additional file 1: Figure S3. **(C)** The level of the biomass *M*. **(D)** Lag1 phase duration *τ*, as a function of initial bottleneck protein concentration *P*. **(E)** Lag1 phase duration (*τ*), as a function of *P* exponential increase rate *ν*.

This is analogous to the control theory strategy known as bang-bang control. A bang-bang strategy posits that in order to produce a given amount of output in minimal time, invest first in producing the limiting component only, and then switch abruptly to making the other elements in the system. This is in contrast to a different strategy in which one makes all components at a given ratio throughout time. In lag1, cells devote their resources to making specific utilization proteins, and then, in lag2, switch to making ribosomes together with more utilization genes. To ask whether this strategy is optimal for making the most cells at a given time *T* after inoculation, we analyzed a mathematical model based on Baranyi's model for bacterial growth [19]. The model, outlined in Figure 5, provides equations for the production of a bottleneck enzymes *P* which provide the substrates for producing biomass *M* (more details in the Methods section). One seeks the best way to temporally allocate production resources between making *P* and making *M*, in order to double the biomass in minimal time.

The model can be exactly solved, using an optimal control method known as Pontryagin's maximum principle [45,46]. The exact solution shows that the optimal allocation is to first make only *P-* as in lag1, and then switch to making both *P* and *M-* as in lag2 (Figure 5A-C). This conclusion applies to a wide range of models and parameters and is not sensitive to the details of the equations used [46].

The exact solution (Figure 5 and Methods section) suggests that the lag phase duration depends on the initial level of bottleneck enzyme *P*_*0*_. The smaller *P*_*0*_, the longer the lag phase because it is advantageous to spend time to produce *P*_*0*_ in order to increase the subsequent rate of biomass production (more precisely, lag duration depends on *P*_*0*_ logarithmically: *τ ~ ln(1/P*_*0*_*)*). If *P*_*0*_ exceeds a critical amount, *P*_*0C*_, it no longer becomes optimal to have a lag1 phase – the optimal *τ* equals zero (Figure 5D). Instead, the cells immediately begin lag2 and produce biomass. This can explain why for some sugars, such as glucose, lag1 phase is not observed. If there are sufficient glucose utilization enzymes in the initial cells, it is better to skip lag1 and begin growth immediately.

The model has three parameters, two of which can be estimated directly from the data: the exponential growth rate *μ* ~ 0.8 hr^-1^ and the bottleneck protein exponential increase rate *ν* ~ 0.5 hr^-1^. From these, and the observed duration of lag1 phase, *τ* ~ 3 hr, one can estimate the initial bottleneck enzyme level in the arabinose experiment: using the formula $\tau =\frac{1}{\nu \;}\mathrm{In}\left(\frac{\nu \;\mu }{P_{0}\mathrm{In}\left(2\right)}\right)$, indicating a low initial enzyme concentration of *P*_*0*_ ~ 0.1 (in units of its effective Michaelis-Menten constant).

Finally, the model also suggests how different individual cells might vary in their lag1 phase duration. It is known that individual cells differ in the concentration of any given protein, an effect known as protein expression noise [22-25]. Cell-cell variations in the initial bottleneck enzyme level *P*_*0*_ will thus result in different lag1 durations. Usually, the distribution of protein concentrations across cells is well described by a Gamma distribution [47], which resembles quite closely a log-normal distributions when protein copy number is not too low. Indeed, our flow cytometry measurements of the *araB* reporter strain show approximately log-normal distributions at early times (SI, Additional file 1: Figure S2). Because lag phase duration goes as the logarithm of *P*_*0*_, the model predicts that cell-cell variation in lag duration will roughly follow a normal distribution (because the lognormal distribution of *P*_*0*_ mean a normal distribution of ln(*P*_*0*_)). This prediction agrees with observed distributions of lag times [26,48].

## Discussion

We found that the seemingly inert lag1 phase shows exponentially increasing activity of a few specific promoters. To observe this, we developed an assay using *E coli* reporter strains, which were imaged during flow cytometry, to measure promoter activity in lag phase. Using this experimental system, we were able to follow cell size and promoter activity dynamics over the bacterial growth curve. This allowed clear distinction between the two lag sub-phases: lag1 and lag2.

With arabinose as the carbon source, we found that gene expression in lag1 phase (the phase where biomass does not grow) is very different from gene expression in exponential phase and lag2 phase (the phase in which biomass grows but not cell number). It is also different from expression in stationary phase before inoculation. In lag1, the genes needed to utilize the specific carbon source in the medium (arabinose) are expressed with exponentially increasing dynamics, despite the fact that biomass does not measurably increase. The exponential rate of expression in lag1 phase is nearly 2-fold slower than the exponential rate of cell growth in exponential phase. Utilization genes for other sugars are not expressed. General growth genes such as ribosomal genes are not expressed in lag1 phase, and begin to be expressed only in lag2 when cell biomass increases. Amino acid biosynthesis genes are also not expressed in lag1 phase on arabinose. In a screen of 140 diverse promoters, the only other promoter that rises sharply above its initial level is the oxidative stress promoter *katG*. The resulting picture resembles the engineering principle of bang-bang control [49-51], in which resources are devoted exclusively at first to the bottleneck proteins to provide carbon and energy for growth, and only later, at lag2, expression switches to a full growth program [52].

One may interpret the exponential rise in expression of utilization genes in lag1 phase by considering a positive feedback process. After inoculation from stationary phase, the cell contains very few transporters and utilization genes for sugars such as arabinose. The influx of arabinose into the cell is low, and ribosomal production is prevented by the stringent response system [53-56]. The cells seem to utilize their meager initial resources [57] to primarily make new utilization genes: arabinose transporters and catabolic enzymes. In turn, due to these transporters and catabolic genes, arabinose influx increases, expression increases in turn, and an auto-catalytic process occurs. The exponential rate of this process is presumably determined by the rate constants of the transporters and enzymes, and the elongation rate of the translation machinery at low nutrient levels. When the cell has enough amino acids to unlock stringent response, ribosomal production ensues together with biomass growth and exit from lag1 (this is in line with the finding of no apparent effect of a *ΔrelA ΔspoT* double deletion on lag2 in rich medium [34], suggesting that ppGpp control of ribosome production is released in lag2).

A very short lag1 phase is expected to be found in fresh media in which cells already express the relevant utilization genes at high levels in stationary phase. For these sugars, such as glucose in the present study, the cell comes prepared for almost immediate growth. It would be of interest to study the evolutionary circumstances that lead cells to choose in which sugar systems to invest during stationary phase.

These findings relate also to theoretical models for lag phase dynamics, such as Baranyi's model [19], which are widely used in the food safety field to estimate lag phase duration in a given food product. Baranyi's model assumes that the cells in lag1 produce a set of enzymes *P* at an exponential rate *ν*, which provide building blocks for biomass production. The present findings may be interpreted as identifying the enzymes *P* as the sugar utilization genes (*ara* genes in the present arabinose medium). In many implementations of Baranyi's model to describe population growth curves, it is assumed for simplicity that the exponential rise in the enzymes *P* occurs at the same rate as the later exponential increase in biomass denoted *μ* (that is, that *ν* = *μ*) [19,58]. The present finding suggest that in the case of arabinose, *P* rises exponentially but at a different (slower) rate than the exponential rise in cell number (*ν < μ*). This finding can lead to refinements of modeling for better predictions of lag phase duration.

The abrupt switch from lag1 with its specific expression program to lag2 with its general growth program may be interpreted in light of optimal control theory. Under a wide range of conditions, in order to achieve the greatest final product (cells), it is optimal to first make only the limiting resources in lag1 (bottleneck proteins that utilize the carbon source), and then abruptly switch to making all of the biomass production machinery in lag2 (Figure 5). Mechanistically, this switch might be implemented by the unlocking of stringent control. A similar strategy is found in wasps that need to allocate resources between making workers and making reproductive individuals [49-51]. Wasps first make only workers, which bring in nutrients, and then, a few weeks before winter, switch to making reproductives (rather than making both workers and reproductives from the onset at a given ratio). Another example is found in development of the mouse gut, where stem cells make an abrupt transition between symmetric and asymmetric divisions, which has been suggested to be the fastest way to reach the desired number of differentiated cells [59].

Future work can extend the present approach to more genes, to fully map the expression strategy in lag phase. It would be important to develop further methods to measure protein quantities required to start the division cycle, in order to develop mechanistic models for bacterial growth phases. Testing the role of stringent response in lag1 phase, and evolutionary experiments to change lag phase duration [60] can help identify genes and regulation important for this process. Another interesting topic is the role of cell-cell variation in lag phase: stochastic effects will result in different individual bacteria making the bang-bang switch at different times [26]. Such studies will require following individual cells and their promoter activity over time.

## Conclusions

In this study we developed a method to explore *E. coli* gene expression in lag phase. We distinguish between lag1 phase in which there is no visible growth, and lag2 in which cells grow but have not yet divided. When arabinose is the sole carbon source, the vast majority of promoters are inactive in lag1, except for the promoters for the carbon source in the medium (arabinose) which are activated with exponentially increasing dynamics. In lag2, *E. coli* switches abruptly to a full growth program of promoter activity. This behavior is consistent with a bang-bang control strategy of allocating resources to making bottleneck components first in order to optimize biomass production over time.

## Methods

### Strains and plasmids

*E. coli* GFP reporter strains (K12 MG1655, with a pUA66 based reporter plasmid, sc101 ori, kan^R^, with the *gfpmut2* gene [61]) are from the fluorescent reporter library described in [35]. Strain U66 with a promoterless reporter plasmid was used for fluorescence background measurements [35,37,62]. All strains are available from Open Biosystems (Thermo Fisher Scientific Molecular Biology, USA).

### Growth conditions and samples preparation

The variability in the history of bacterial populations can lead to variability in lag phase behavior even within the same strain [63-65]. We thus employed procedures to reduce lag phase variability between biological repeats and within each experiment. Media was filter sterilized using 0.2-μm pore size filters (FP 30/0,2 CA-S, Whatman, UK; and Fast PES filter unit, Nalgene, Thermo Fisher Scientific, USA) to reduce false-positive events in the flow cytometry experiments, and to avoid autoclave steps that can introduce variations in pH and in other factors to the medium [34,66,67]. The same batches of medium constituents were used in all experiments to minimize experimental error. All liquid handling and incubation steps were carried out using a robotic liquid handler (Freedom Evo, Tecan, Switzerland) and incubator (StoreX STX44 ICBT, Liconic Instruments, Liechtenstein) system. Incubations with shaking (80 Hz = 4800 RPM) were carried out at 37°C. Strains from frozen stocks were pre-grown for 48 hours in u-shaped bottom 2 ml 96-deepwell plates (PlateOne, USA Scientific, USA), with 600 μl M9 minimal medium containing 0.2% glucose, 0.05% casamino acids, and 50 μg/ml kanamycin. This relatively long incubation time ensures that the cells are in stationary phase long enough to reduce the effects of cell history, and leads to a sizable lag phase. Longer incubation times increase the lag phase cell-to-cell variability presumably due to increased stress [20,26,48,63,64,68]. We controlled for different amounts of time spent in stationary phase pre-inoculation, finding that this variable had little effect on the present conclusions.

The deep well plates were covered with aeration-permitting lids (Universal Lid, Seahorse Bioscience, USA), to reduce contamination and evaporation. The experimental deep well plates were prepared with M9 minimal medium (final volume 500 μl) containing 50 μg/ml kanamycin and 0.2% of a certain carbon source (glucose; L-arabinose). The glucose medium also contained 0.05% casamino acids (Bacto, BD, USA).

During 15 hours, every 5–180 minutes, a different column was inoculated with 8 reporter strains at a 1:750 dilution from the stationary phase culture. After 15 hours, the deep well plates contained cells that were from 5 minutes up to 15 hours in fresh medium. This conveniently generated a full time-course of growth in one plate, that could be analyzed in a single flow cytometry session. One column in each plate was not inoculated and was used for both medium contamination detection and blank sample measurements. Reporter strains from the stationary phase cultures were diluted 1:750 into pre-cooled M9, and represented the strains at t = 0 (end of stationary phase or beginning of lag phase). After the experimental incubation, the cells were rapidly cooled to 4°C, diluted according to their density (from no dilution at all, up to 1:100 dilution), to avoid coincidence in the flow cytometry instruments [69,70], and transferred into pre-cooled u-shaped 96-wells plates (Costar, Corning, USA) for the automated flow cytometry analysis. All samples were measured at most 4 hours after the growth experiments ended. The same samples were analyzed both using the LSRII and the ImageStream^X^ instruments.

No fixation or staining were used as these procedures potentially affect the side scatter (SSC), forward scatter (FSC), and GFP values of bacterial cells, as well as the bacterial count [70-72]. Moreover, the added steps necessary for these procedures might increase variability between the samples and between the experiments.

### Flow cytometry measurements

#### Standard flow cytometry

20 μl sample at a flow rate of 1 μl/s were analyzed per sample using a LSRII (BD, USA) flow cytometry instrument (settings: FSC 648 mV, SSC 346 mV, GFP 450 mV, threshold collection FSC > 200) at 4°C. The cells' FSC, SSC and GFP values were collected. The events rate for the bacterial samples was between 1,000-9,500 events/s, and did not come near the upper limit of the instrument (20,000 events/s), to avoid coincidence [69,70]. A total of 20,000-190,000 events were collected per sample. Blank samples with medium only were used in each experiment to exclude contaminations, and to calculate background event numbers, with an average event rate of 50 events/s (Additional file 1: Figure S5). FSC & SSC were used for gating. In contrast to most flow cytometry experiments that usually use narrow gating to achieve measurements of a homogenized population, a wide gating was used in this study (Additional file 1: Figure S6). This large gating allowed measuring the entire population, and especially to monitor cell size and cell number. The number of background events in the gated area was negligible, between 0.3-3% of the total number of gated events per sample. The mean day-to-day error between fluorescence measurements for the arabinose system reporters was 9%, and below 17% for cell count.

Bacterial growth curves were calculated by the following procedure: In each well, the same liquid volume and liquid flow rate were used to count the number of events (cells) [73]. Combined with the known dilution coefficients (from the original experimental deep well plates), we calculated bacterial cell counts at each time point for each reporter strain.

#### Imaging flow cytometry

Around 4000 events were collected per sample using the ImageStream^X^ imaging flow cytometry instrument and Inspire software (Amnis, Seatle, USA). The cells' bright field (BF), SSC & GFP images were taken at an event rate of 5–500 cells/s. The instrument uses beads to track the cell flow and to focus on the cells. Bead images were ignored during image acquisition or removed from the analysis to increase image analysis accuracy. Instrument settings for image collection: *raw_max_pixel_SSC* < 200 (ignored most of the bead images), *raw_max_pixel_GFP* < 4094 (ignored saturated GFP images), *raw_min_area_BF* > 1 square μm (>9 pixels, this removed small objects suspected as dust and cell fragments). Image analysis was performed using ImageStream^X^ analysis software IDEAS 4.0 (Amnis, Seatle, USA). Gating: more beads were removed by gating with *mean_pixel_BF* and *mean_pixel_SSC*, unfocused cells were removed by gating using *gradient_RMS_BF*, and more beads were removed by gating with *aspect_ratio_intensity_BF* and *min_pixel_BF*[74]. Flow aligns the cell long axis with the flow direction, so that length measurements in 2D images are an accurate indication of true length [75,76]. Measurements of mean cell length had a mean day to day error of 3%.

Standard flow cytometry and imaging flow cytometry analysis were conducted on the same samples. All experiments were repeated at least twice.

#### Optimal control model

We model the lag phase enzyme dynamics using the framework of Baranyi and Roberts [19]. The cells make biomass *M* at a rate that depends on a bottleneck enzyme (representing for example transporters and catabolic enzymes for arabinose utilization) whose concentration is *P*. The rate of biomass production is dependent on *P* in a Michaelis-Menten form:

(1) $$\frac{\mathrm{dM}}{\mathrm{dt}}=u\;\mu \frac{P}{\left(1+P\right)}M$$

Where *μ* is the maximal growth rate, and *P* is given in units of its effective halfway concentration, so that at *P* = 1 biomass production is half-maximal. The function *u*(*t*) is the proportion of resources that the cell devotes to biomass production, which can vary between one and zero.

The bottleneck enzyme *P* is produced at rate *νP*, times the fraction of resources the cell devotes to it, namely 1-*u:*

(2) $$\frac{\mathrm{dP}}{\mathrm{dt}}=\left(1-u\right)\;\nu \;P$$

The objective in the optimization problem we study is to divide rapidly in the new condition. We thus seek the control profile *u*(*t*) that will double the biomass in the minimal amount of time, *T*. Initial conditions are *P*(*t* = 0) = *P*_*0*_, the amount of enzyme present in the inoculated cells, and initial biomass *M*(*t* = 0) = *M*_*0*_.

According to Pontryagin’s maximum principle [45,46], such minimal time problems are solved by the profile *u*(*t*) that maximizes at every time point the Hamiltonian:

(3) $$\begin{array}{l} H=-1+\lambda_{1}\frac{\mathrm{dM}}{\mathrm{dt}}+\lambda_{2}\frac{\mathrm{dP}}{\mathrm{dt}}\\ \;=-1+\lambda_{1}\;u\;\mu \frac{P}{\left(1+P\right)}M+\lambda_{2}\left(1-u\right)\;\nu \;P \end{array}$$

The Lagrange multipliers *λ*_*1*_(*t*) and *λ*_*2*_(*t*) follow Hamilton’s equations:

(4) $$\frac{d\;\lambda_{1}}{\mathrm{dt}}=-\frac{\partial H}{\partial M},\frac{d\;\lambda_{2}}{\mathrm{dt}}=-\frac{\partial H}{\partial P}$$

Because the Hamiltonian *H* is linear in *u* (Eq 3), it can be maximal only at the extreme values that *u* can take, namely zero and one. This means bang-bang control: devote all the cell resources to either *M* or *P*, with sharp switches between these states allowed. Bang-Bang control is expected with any Hamiltonian function that is linear or convex in *u*, a very wide class of possible models [46]. Analyzing the equations for *λ*_*1*_ and *λ*_*2*_ shows at most a single switch between the extreme values of *u* can occur in the present model.

Assuming a single switch between making only *P* and then switching to make only *M*, with switching at time *τ*, one can solve Eq 2 with *u* = 0 to find

(5) $$P\left(\tau \right)=P_{0}e^{\nu \;\tau }$$

From time *τ* to the division time *T*, one has *u* = 1, so *P* is constant = *P*(τ) and *M* increases exponentially:

(6) $$M\left(T\right)=M_{0}e^{\mu \frac{\left(P\left(\tau \right)\right)}{\left(1+P\left(\tau \right)\right)}\left(T-\tau \right)}$$

We seek the time *T* where biomass doubles, *M*(*T*) = 2 *M*_*0*_. Solving for *T* results in

(7) $$T=\tau +\frac{In\left(2\right)}{\mu }\left(1+\frac{1}{P_{0}e^{\nu \;\tau }}\right)$$

Finding the lag duration *τ* that minimizes the time to first division *T*, namely d*T*/d*τ* = 0, results in the following solution:

(8) $$\tau =\frac{1}{\nu }\;\mathrm{In}\left(\frac{\nu }{\nu_{c}}\right)$$

where

(9) $$\nu_{c}=\frac{\mu \;P_{0}}{\mathrm{In}\left(2\right)}$$

Thus, *τ* is positive only when *ν* > *ν*_*c*_. It is equal to zero for *ν* < *ν*_*c*_, meaning no lag1 phase and a direct transition to lag2 (Figure 5E). The lag phase duration *τ* is maximal when *ν* = *e · ν*_*c*_, where *e* = 2.71. Another way to consider this result is that for a given *ν* and *μ*, there is a critical initial enzyme level *P*_*0*_ above which there is no lag1: *P*_*0C*_ = ln(2)*ν*/*μ*. For the present arabinose parameters, *P*_*0C*_ ~ 0.4. The duration of lag2 in this model is log(2)/*μ* + 1/*ν* ~ 3 h, which is shorter than the observed arabinose lag2 phase duration, ~5 h. This discrepancy might be explained by using a stochastic model in which lag phase differs between cells. Note that the experimental data (Figure 3) shows increases of total *P* in lag2 (fluorescence of the *ara* promoters), whereas the model shows that *P* concentration becomes constant at lag2. These two observations are consistent, because biomass grows in lag2, and therefore constant *P* concentration means an increase in total *P* protein as observed in the experiment.

## Abbreviations

BF: Bright field; CFU: Colony forming unit; FC: Flow cytometry; FSC: Forward scatter; GFP: Green fluorescent protein; M9CGLU: M9 + 0.05% casamino acids + 0.2% glucose minimal medium; M9ARA: M9 + 0.2% arabinose minimal medium; OD: Optical density; SSC: Side scatter.

## Competing interests

The authors declare that they have no competing interests.

## Authors’ contributions

DM, AB and UA designed research; DM, ED and AZ conducted research; DM, ED, ZP and UA analyzed data; DM, ED and UA wrote the paper. All authors read and approved the final manuscript.

## Supplementary Material

Additional file 1 **Supplemental information.** Supporting figures and supporting tables. Click here for file

Additional file 2 **Screen data.** 140 promoters normalized screen data. Click here for file

Additional file 3 **Screen plots.** 140 promoters normalized screen data plots. Click here for file

## References

[B1] Müller M Ueber den Einfluss von Fieber temperaturen auf die Wachstumsgeschwindigkeit und die Virulenz des Typhus Bacillus Z Hyg Infektionskr 1895 7 245

[B2] Penfold WJ On the nature of bacterial lag J Hyg (Lond) 1914 7 215 241 20474577 10.1017/s0022172400005817 PMC2206768

[B3] Monod J The growth of bacterial cultures Annu Rev Microbiol 1949 7 371 394 10.1146/annurev.mi.03.100149.002103

[B4] Swinnen IAM Bernaerts K Dens EJJ Geeraerd AH Van Impe JF Predictive modelling of the microbial lag phase: a review Int J Food Microbiol 2004 7 137 159 10.1016/j.ijfoodmicro.2004.01.006 15193801

[B5] Koyuncu S Andersson MG Häggblom P Accuracy and sensitivity of commercial PCR-based methods for detection of salmonella enterica in feed Appl Environ Microbiol 2010 7 2815 2822 10.1128/AEM.02714-09 20228106 PMC2863422

[B6] Van Impe J McMeekin T Olley J Ratkowsky D 3rd international conference on predictive modeling in foods Int J Food Microbiol 2002 7 107 454 10.1016/S0168-1605(01)00642-0

[B7] Bättig P Hathaway LJ Hofer S Mühlemann K Serotype-specific invasiveness and colonization prevalence in Streptococcus pneumoniae correlate with the lag phase during in vitro growth Microbes Infect 2006 7 2612 2617 10.1016/j.micinf.2006.07.013 16938479

[B8] Hathaway LJ Brugger SD Morand B Bangert M Rotzetter JU Hauser C Graber WA Gore S Kadioglu A Mühlemann K Capsule type of Streptococcus pneumoniae determines growth phenotype PLoS Pathog 2012 7 e1002574 10.1371/journal.ppat.1002574 22412375 PMC3297593

[B9] Frimodt-Møller N Sebbesen O Frølund Thomsen V The pneumococcus and the mouse protection test: importance of the lag phase in vivo Chemotherapy 1983 7 128 134 10.1159/000238186 6839864

[B10] Baranyi J George SM Kutalik Z Parameter estimation for the distribution of single cell lag times J Theor Biol 2009 7 24 30 10.1016/j.jtbi.2009.03.023 19328813

[B11] Buchanan RL Cygnarowicz ML A mathematical approach toward defining and calculating the duration of the lag phase Food Microbiol 1990 7 237 240 10.1016/0740-0020(90)90029-H

[B12] Buchanan RL Solberg M Interaction of sodium nitrate, oxygen and ph on growth of staphylococcus aureus J Food Sci 1972 7 81 85 10.1111/j.1365-2621.1972.tb03391.x

[B13] McKellar RC Knight K A combined discrete-continuous model describing the lag phase of Listeria monocytogenes Int J Food Microbiol 2000 7 171 180 10.1016/S0168-1605(99)00204-4 10777067

[B14] Pirt SJ Principles of microbe and cell cultivation 1975 New York: Wiley

[B15] Zhou K George SM Métris A Li PL Baranyi J Lag phase of salmonella enterica under osmotic stress conditions Appl Environ Microbiol 2011 7 1758 1762 10.1128/AEM.02629-10 21193660 PMC3067304

[B16] Zwietering MH Rombouts FM van ’t Riet K Comparison of definitions of the lag phase and the exponential phase in bacterial growth J Appl Bacteriol 1992 7 139 145 10.1111/j.1365-2672.1992.tb01815.x 1556037

[B17] Baranyi J Roberts TA McClure P A non-autonomous differential equation to modelbacterial growth Food Microbiol 1993 7 43 59 10.1006/fmic.1993.1005

[B18] Srivastava AK Volesky B Characterization of transient cultures of clostridium acetobutylicum Biotechnol Prog 1990 7 408 420 10.1021/bp00006a002

[B19] Baranyi J Roberts TA A dynamic approach to predicting bacterial growth in food Int J Food Microbiol 1994 7 277 294 10.1016/0168-1605(94)90157-0 7873331

[B20] Pin C Baranyi J Kinetics of single cells: observation and modeling of a stochastic process Appl Environ Microbiol 2006 7 2163 2169 10.1128/AEM.72.3.2163-2169.2006 16517667 PMC1393193

[B21] Métris A Le Marc Y Elfwing A Ballagi A Baranyi J Modelling the variability of lag times and the first generation times of single cells of E. coli Int J Food Microbiol 2005 7 13 19 10.1016/j.ijfoodmicro.2004.10.004 15854688

[B22] Elowitz MB Levine AJ Siggia ED Swain PS Stochastic gene expression in a single cell Science 2002 7 1183 1186 10.1126/science.1070919 12183631

[B23] Locke JCW Young JW Fontes M Jiménez MJH Elowitz MB Stochastic pulse regulation in bacterial stress response Science 2011 7 366 369 10.1126/science.1208144 21979936 PMC4100694

[B24] Kaern M Elston TC Blake WJ Collins JJ Stochasticity in gene expression: from theories to phenotypes Nat Rev Genet 2005 7 451 464 10.1038/nrg1615 15883588

[B25] Golding I Paulsson J Zawilski SM Cox EC Real-time kinetics of gene activity in individual bacteria Cell 2005 7 1025 1036 10.1016/j.cell.2005.09.031 16360033

[B26] Elfwing A LeMarc Y Baranyi J Ballagi A Observing growth and division of large numbers of individual bacteria by image analysis Appl Environ Microbiol 2004 7 675 678 10.1128/AEM.70.2.675-678.2004 14766541 PMC348858

[B27] Larsen N Boye M Siegumfeldt H Jakobsen M Differential expression of proteins and genes in the Lag phase of lactococcus lactis subsp. Lactis grown in synthetic medium and reconstituted skim milk Appl Environ Microbiol 2006 7 1173 1179 10.1128/AEM.72.2.1173-1179.2006 16461664 PMC1392913

[B28] Cuny C Lesbats M Dukan S Induction of a global stress response during the first step of Escherichia coli plate growth Appl Environ Microbiol 2007 7 885 889 10.1128/AEM.01874-06 17142356 PMC1800750

[B29] Novotna J Vohradsky J Berndt P Gramajo H Langen H Li X-M Minas W Orsaria L Roeder D Thompson CJ Proteomic studies of diauxic lag in the differentiating prokaryote Streptomyces coelicolor reveal a regulatory network of stress-induced proteins and central metabolic enzymes Mol Microbiol 2003 7 1289 1303 10.1046/j.1365-2958.2003.03529.x 12787356

[B30] Osuna R Lienau D Hughes KT Johnson RC Sequence, regulation, and functions of fis in salmonella typhimurium J Bacteriol 1995 7 2021 2032 7536730 10.1128/jb.177.8.2021-2032.1995 PMC176845

[B31] McKellar RC Correlation between the change in the kinetics of the ribosomal RNA rrnB P2 promoter and the transition from lag to exponential phase with pseudomonas fluorescens Int J Food Microbiol 2008 7 11 17 10.1016/j.ijfoodmicro.2007.10.003 18036694

[B32] McKellar RC Effect of starvation on expression of the ribosomal RNA rrnB P2 promoter during the lag phase of pseudomonas fluorescens Int J Food Microbiol 2007 7 307 315 10.1016/j.ijfoodmicro.2006.09.022 17169452

[B33] McKellar RC Effect of sub-lethal heating and growth temperature on expression of the ribosomal RNA rrnB P2 promoter during the lag phase of pseudomonas fluorescens Int J Food Microbiol 2007 7 248 259 10.1016/j.ijfoodmicro.2007.01.009 17368596

[B34] Rolfe MD Rice CJ Lucchini S Pin C Thompson A Cameron ADS Alston M Stringer MF Betts RP Baranyi J Peck MW Hinton JCD Lag phase is a distinct growth phase that prepares bacteria for exponential growth and involves transient metal accumulation J Bacteriol 2012 7 686 701 10.1128/JB.06112-11 22139505 PMC3264077

[B35] Zaslaver A Bren A Ronen M Itzkovitz S Kikoin I Shavit S Liebermeister W Surette MG Alon U A comprehensive library of fluorescent transcriptional reporters for Escherichia coli Nat Methods 2006 7 623 628 10.1038/nmeth895 16862137

[B36] Kaplan S Bren A Zaslaver A Dekel E Alon U Diverse two-dimensional input functions control bacterial sugar genes Mol Cell 2008 7 786 792 10.1016/j.molcel.2008.01.021 18374652 PMC2366073

[B37] Madar D Dekel E Bren A Alon U Negative auto-regulation increases the input dynamic-range of the arabinose system of Escherichia coli BMC Syst Biol 2011 7 111 10.1186/1752-0509-5-111 21749723 PMC3163201

[B38] Hudson JA Mott SJ Comparison of lag times obtained from optical density and viable count data for a strain of pseudomonas fragi J Food Saf 1994 7 329 339 10.1111/j.1745-4565.1994.tb00604.x

[B39] Robinson TP Ocio MJ Kaloti A Mackey BM The effect of the growth environment on the lag phase of Listeria monocytogenes Int J Food Microbiol 1998 7 83 92 10.1016/S0168-1605(98)00120-2 9849786

[B40] Kell DB Young M Bacterial dormancy and culturability: the role of autocrine growth factors Curr Opin Microbiol 2000 7 238 243 10.1016/S1369-5274(00)00082-5 10851153

[B41] Weichart DH Kell DB Characterization of an autostimulatory substance produced by Escherichia coli Microbiology 2001 7 1875 1885 11429464 10.1099/00221287-147-7-1875

[B42] Mukamolova GV Kaprelyants AS Young DI Young M Kell DB A bacterial cytokine Proc Natl Acad Sci U S A 1998 7 8916 8921 10.1073/pnas.95.15.8916 9671779 PMC21177

[B43] Kempner ES Hanson FE Aspects of light production by photobacterium fischeri J Bacteriol 1968 7 975 979 5643069 10.1128/jb.95.3.975-979.1968 PMC252118

[B44] Turovskiy Y Kashtanov D Paskhover B Chikindas ML Quorum sensing: fact, fiction, and everything in between Adv Appl Microbiol 2007 7 191 234 17869606 10.1016/S0065-2164(07)62007-3 PMC2391307

[B45] Pontryàgin LS Boltyanskii VG Gamkrelidze RV Mishchenko EF The mathematical theory of optimal processes 1962 New York: John Wiley

[B46] Alexander RM Optima for animals 1996 Princeton: Princeton University Press

[B47] Cai L Friedman N Xie XS Stochastic protein expression in individual cells at the single molecule level Nature 2006 7 358 362 10.1038/nature04599 16541077

[B48] Levin-Reisman I Gefen O Fridman O Ronin I Shwa D Sheftel H Balaban NQ Automated imaging with ScanLag reveals previously undetectable bacterial growth phenotypes Nat Methods 2010 7 737 739 10.1038/nmeth.1485 20676109

[B49] Ishay J Bytinski-Saltz H Shulov A Contributions to the bionomics of the oriental hornet Vespa orientalis Isr J Entomol 1967 7 45 106

[B50] Macevicz S Oster G Modeling social insect populations II: optimal reproductive strategies in annual eusocial insect colonies Behav Ecol Sociobiol 1976 7 265 282 10.1007/BF00300068

[B51] Oster GF Wilson EO Caste and ecology in the social insects. (Mpb-12) 1979 Princeton: Princeton University Press 740003

[B52] Shoval O Sheftel H Shinar G Hart Y Ramote O Mayo A Dekel E Kavanagh K Alon U Evolutionary trade-offs, pareto optimality, and the geometry of phenotype space Science 2012 7 1157 1160 10.1126/science.1217405 22539553

[B53] Cashel M Kalbacher B The control of ribonucleic acid synthesis in Escherichia coli. V. Characterization of a nucleotide associated with the stringent response J Biol Chem 1970 7 2309 2318 4315151

[B54] Cashel M Gentry DR Hernandez VH Vinella D Ingraham JL, Neidhardt FC, Ingraham JL, Neidhardt FC The stringent response Escherichia coli & salmonella typhimurium: cellular & molecular biology. Volume 1 1996 2 Washington DC: ASM Press 1458 1496

[B55] Schneider DA Ross W Gourse RL Control of rRNA expression in Escherichia coli Curr Opin Microbiol 2003 7 151 156 10.1016/S1369-5274(03)00038-9 12732305

[B56] Bouveret E Battesti A The stringent response. In Bacterial stress response. 2nd Edition. Edited by Storz G, Hengge R 2011 Washington, DC: ASM Press

[B57] Yamamotoya T Dose H Tian Z Fauré A Toya Y Honma M Igarashi K Nakahigashi K Soga T Mori H Matsuno H Glycogen is the primary source of glucose during the lag phase of E. coli proliferation Biochim Biophys Acta 1824 7 1442 1448 10.1016/j.bbapap.2012.06.010 22750467

[B58] Zhou K George SM Li PL Baranyi J Effect of periodic fluctuation in the osmotic environment on the adaptation of Salmonella Food Microbiol 2012 7 298 302 10.1016/j.fm.2011.09.016 22265315

[B59] Itzkovitz S Blat IC Jacks T Clevers H van Oudenaarden A Optimality in the development of intestinal crypts Cell 2012 7 608 619 10.1016/j.cell.2011.12.025 22304925 PMC3696183

[B60] Oxman E Alon U Dekel E Defined order of evolutionary adaptations: experimental evidence Evolution 2008 7 1547 1554 10.1111/j.1558-5646.2008.00397.x 18410537

[B61] Cormack BP Valdivia RH Falkow S FACS-optimized mutants of the green fluorescent protein (GFP) Gene 1996 7 1 Spec No 33 38 8707053 10.1016/0378-1119(95)00685-0

[B62] Hart Y Madar D Yuan J Bren A Mayo AE Rabinowitz JD Alon U Robust control of nitrogen assimilation by a bifunctional enzyme in E Coli. Mol Cell 2011 7 117 127 10.1016/j.molcel.2010.12.023 21211727

[B63] Hershey AD Factors limiting bacterial growth J Bacteriol 1939 7 285 299 16560205 10.1128/jb.37.3.285-299.1939 PMC374464

[B64] Pin C Baranyi J Single-cell and population lag times as a function of cell age Appl Environ Microbiol 2008 7 2534 2536 10.1128/AEM.02402-07 18296533 PMC2293159

[B65] D’Arrigo M de Fernando GDG Velasco de Diego R Ordóñez JA George SM Pin C Indirect measurement of the lag time distribution of single cells of listeria innocua in food Appl Environ Microbiol 2006 7 2533 2538 10.1128/AEM.72.4.2533-2538.2006 16597954 PMC1449042

[B66] Biesta-Peters EG Mols M Reij MW Abee T Physiological parameters of Bacillus cereus marking the end of acid-induced lag phases Int J Food Microbiol 2011 7 42 47 10.1016/j.ijfoodmicro.2011.04.024 21592605

[B67] Gennis RB Stewart V Ingraham JL, Neidhardt FC Respiration Escherichia Coli & Salmonella Typhimurium: Cellular & Molecular Biology. Volume 1 2 Washington, DC: ASM Press 217 261

[B68] Guillier L Pardon P Augustin J-C Influence of stress on individual lag time distributions of listeria monocytogenes Appl Environ Microbiol 2005 7 2940 2948 10.1128/AEM.71.6.2940-2948.2005 15932988 PMC1151854

[B69] Marie D Vaulot D Partensky F Application of the novel nucleic acid dyes YOYO-1, YO-PRO-1, and PicoGreen for flow cytometric analysis of marine prokaryotes Appl Environ Microbiol 1996 7 1649 1655 8633863 10.1128/aem.62.5.1649-1655.1996 PMC167939

[B70] Gasol JM del Giorgio PA Using flow cytometry for counting natural planktonic bacteria and understanding the structure of planktonic bacterial communities Sci Mar 2000 7 197 224

[B71] Kamiya E Izumiyama S Nishimura M Mitchell JG Kogure K Effects of fixation and storage on flow cytometric analysis of marine bacteria J Oceanogr 2007 7 101 112 10.1007/s10872-007-0008-7

[B72] Günther S Hübschmann T Rudolf M Eschenhagen M Röske I Harms H Müller S Fixation procedures for flow cytometric analysis of environmental bacteria J Microbiol Methods 2008 7 127 134 10.1016/j.mimet.2008.05.017 18584902

[B73] Monfort P Baleux B Comparison of flow cytometry and epifluorescence microscopy for counting bacteria in aquatic ecosystems Cytometry 1992 7 188 192 10.1002/cyto.990130213 1547667

[B74] George TC Fanning SL Fitzgerald-Bocarsly P Fitzgeral-Bocarsly P Medeiros RB Highfill S Shimizu Y Hall BE Frost K Basiji D Ortyn WE Morrissey PJ Lynch DH Quantitative measurement of nuclear translocation events using similarity analysis of multispectral cellular images obtained in flow J Immunol Methods 2006 7 117 129 10.1016/j.jim.2006.01.018 16563425

[B75] Rajwa B Venkatapathi M Ragheb K Banada PP Hirleman ED Lary T Robinson JP Automated classification of bacterial particles in flow by multiangle scatter measurement and support vector machine classifier Cytometry Part A 2008 7 369 379 10.1002/cyto.a.20515 18163466

[B76] Yamaguchi N Torii M Uebayashi Y Nasu M Rapid, Semiautomated Quantification of Bacterial Cells in Freshwater by Using a Microfluidic Device for On-Chip Staining and Counting Appl Environ Microbiol 2011 7 1536 1539 10.1128/AEM.01765-10 21169431 PMC3067242

