# Supplementary material for: Promoter activity dynamics in the lag phase of Escherichia coli
Source: BMC Syst Biol. 2013 Dec 30;7:136. doi: 10.1186/1752-0509-7-136 (PMC3918108; doi:10.1186/1752-0509-7-136)
Supplement: Additional file 1 — Supplemental information. Supporting figures and supporting tables. [file 1752-0509-7-136-S1.pdf]

Promoter activity dynamics in the lag phase of *Escherichia coli*

Daniel Madar[1], Erez Dekel[1], Anat Bren[1], Anat Zimmer[1], Ziv Porat[2], Uri Alon[1]

[1]Department of Molecular Cell Biology, The Weizmann Institute of Science, Rehovot, 76100, Israel

[2]Biological Services Unit, The Weizmann Institute of Science, Rehovot, 76100, Israel

Supplemental information

Supplemental Data

Table S1: Lag phase definitions.

Reference	Literature definitions	Present terminology
(Monod, 1949)	Lag phase is the period in a new condition until there is growth in cell density.	Lag1
(Monod, 1949)	Acceleration phase is the phase between the end of lag phase (as in (Monod, 1949)) until maximal growth rate is reached.	Lag2+ beginning of exp. phase
(Buchanan and Solberg, 1972)	Lag phase is the period in a new condition until the cells double their number.	Lag1+ Lag2
(Penfold, 1914; Pirt, 1975)	Lag phase is the period in a new condition until the maximal growth rate is reached.	Lag1+ Lag2+ beginning of exp. phase
(Buchanan and Cygnarowicz, 1990)	Lag phase is the period in a new condition until the maximal acceleration in growth rate.	Lag1+ Lag2+ beginning of exp. phase
(Swinnen et al., 2004; Zwietering et al., 1992)	Lag phase is the period in a new condition until the intersection point between the inoculum level and the growth curve exponential part tangent line.	Lag1+ Lag2
(Srivastava and Volesky, 1990; Baranyi and Roberts,1994)	Lag phase is the period in a new condition, until a critical substance in the cell, reaches a threshold level that enables cell\biomass growth.	Lag1
(McKellar and Knight, 2000)	Lag phase is the period in a new condition that is composed of adaptation time (lag1) plus doubling time (lag2).	Lag1+ Lag2
(Baranyi et al., 2009)	Lag phase is the period in a new condition until the first division (composed of time until volume growth plus generation time- basically the same as (McKellar and Knight, 2000)).	Lag1+ Lag2
(Zhou et al., 2011)	Same as (Baranyi et al., 2009), but redefined as the lag of the surviving subpopulation.	Lag 1+ Lag 2

Table S1. Lag phase definitions. The different lag phase definitions in the literature.

Figure S1: Discrimination between lag1, lag2 and acceleration phases of a single cell

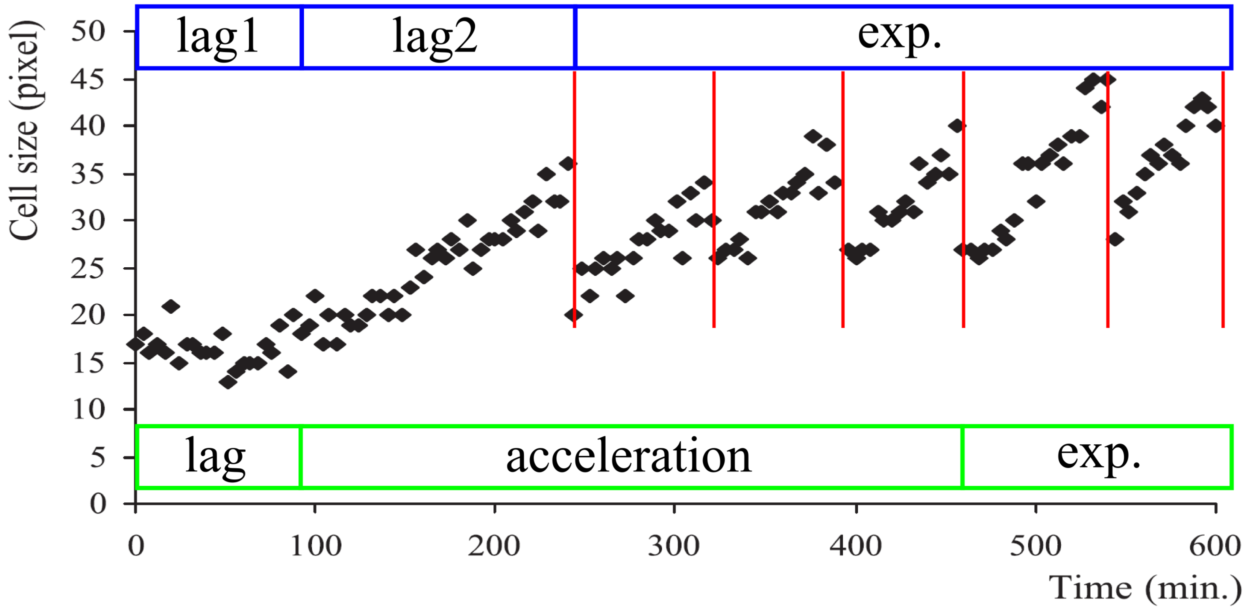

Figure S1. Discrimination between lag1, lag2 and acceleration phases of a single cell. Adopted with permission from Métris et al. (Métris et al., 2005). Number of pixels as a function of time; this cell divided six times during the observation period. The division times are defined as the times before a sudden drop in pixels. Each division event is marked with a red vertical line. The top blue growth phases segmentation is our segmentation (see manuscript for details). The lower green growth phases segmentation is Monod's segmentation (Monod, 1949).

__Figure S2: The araB promoter activity is a graded response__

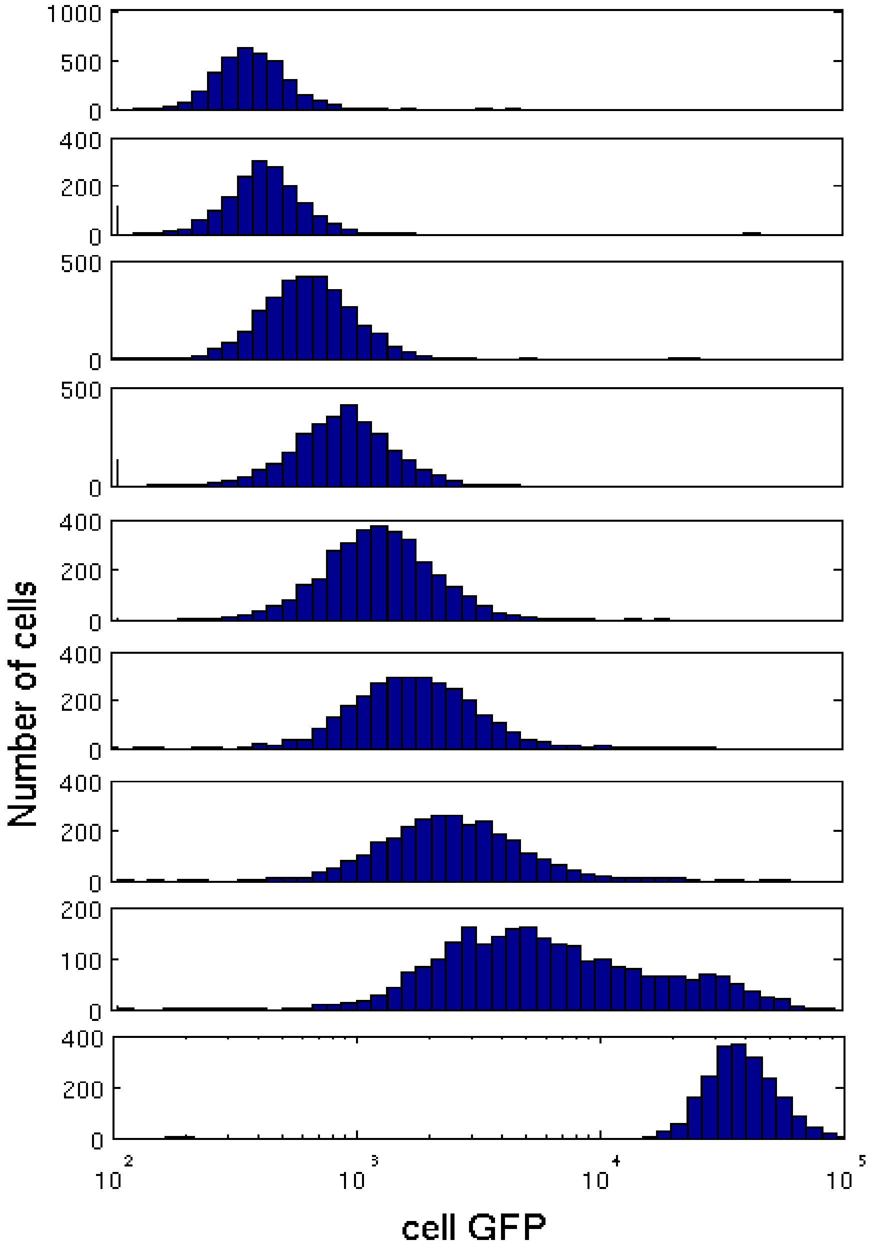

Figure S2. The *araB* promoter activity is a graded response. Histograms of GFP distributions throughout time for the *araB* promoter activity. Time points (hours) from top to bottom panels: 0:00, 0:06, 0:36, 1:06, 2:06, 3:06, 4:06, 6:06, 15:06.

Figure S3: Fitted model to the arabinose promoters results on arabinose minimal medium.

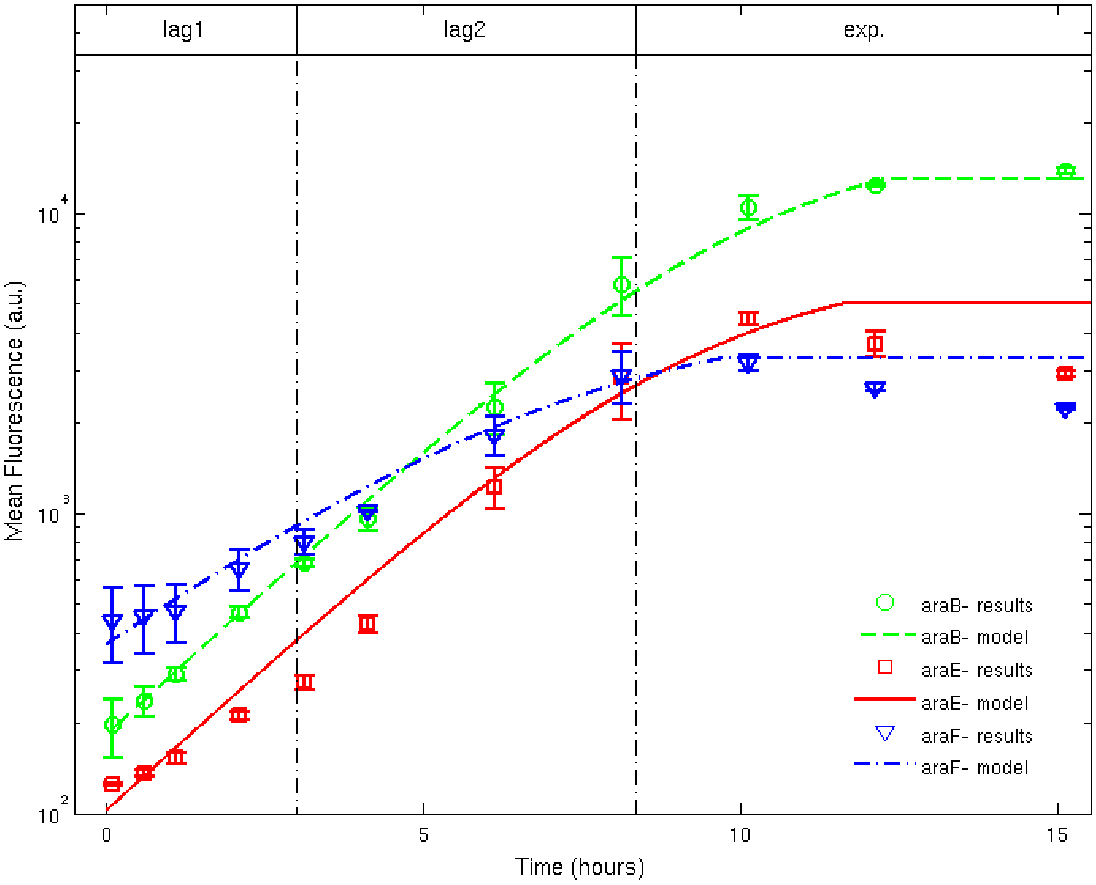

Figure S3. Fitted model to the arabinose promoters results on arabinose minimal medium. Mean GFP fluorescence (arbitrary units) of the cells over time in minimal arabinose medium (M9ARA), for the arabinose system reporter strains, with segmentation into growth phases. The lines are fit to our model (bottleneck genes). The model parameters are: μ=0.76±0.07 hour^{-1} (bacterial browth rate). For *araB:* v=0.45±0.02 hour^{-1},(the specific rate of *araB*) and P_0=1300 (initial GFP value). For *araE:* v=0.44±0.03 hour^{-1} (the specific rate of *araE*), and P_0=750 (initial GFP value). For *araF:* v=0.35±0.02 hour^{-1} (the specific rate of *araF*), and R_0=2600 (initial GFP value).

Screen for promoters activated during lag1:

In order to verify our results, we conducted a screen with an additional 140 promoters of various functions. Only two oxidative stress promoters , *soxS* and *katG* showed more than a three fold increase in lag1. We tested these reporters strains at high temporal resolution (12 time points). In this experiment only *katG* showed the same increase, whereas *soxS* did not, increasing only at lag2.

We also found minor increases in some of the iron transporters and metabolism reporters, similar to Rolfe et. al. (Rolfe et. al. 2012). Note that even a relatively minor increase can be significant when the basal GFP level is high (as the lag2 and exponential phases increase of the ribosomal reporters).

The screen data and plots are in additional files **Madar_AF2_ScreenData.xls** and **Madar_AF3_ScreenPlots.pdf**

Supplemental Experimental Procedures

Figure S4: Comparison of bacterial count methods

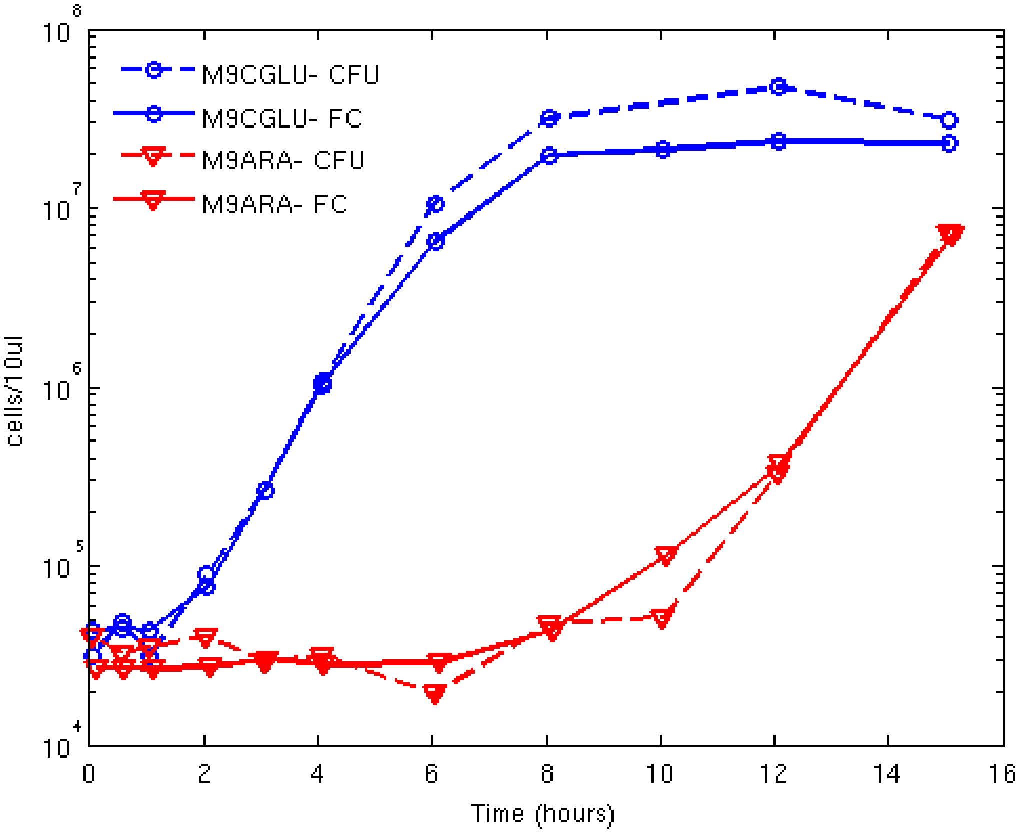

Figure S4 Comparison of bacterial count methods. Bacterial counts over time. The blue lines with blue circles are bacterial counts in M9C+0.2% glucose. The red lines with red triangles are bacterial counts in M9+0.2% arabinose. Solid lines are bacterial counts using flow cytometry (LSRII), and dashed lines are bacterial counts using colony counting of colony forming units (CFU) on LB agar+50 µg/ml kanamycin plates. The measurements for each medium were taken from the same samples for both counting methods. The error for FC counts is 17% and the mean error for CFU counts is 26%. The lines are guide to the eye.

Figure S5: Flow cytometry raw data of a blank sample

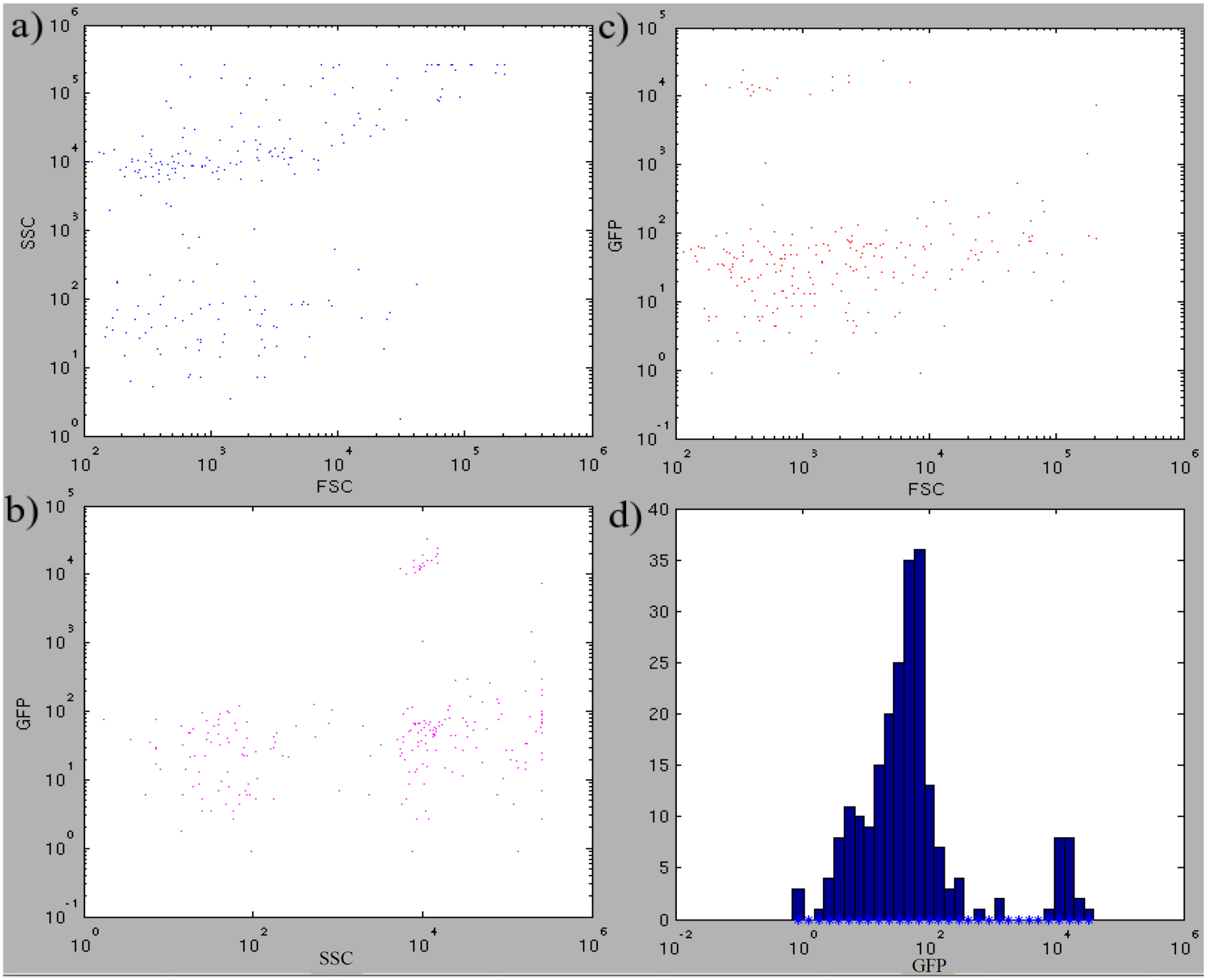

Figure S5. Flow cytometry raw data of a blank sample. An example for raw data analyzed with LSRII from a sample with medium only and no bacteria. (A) SSC against FCS data. (B) GFP against SSC data. (C) GFP against FSC data. (D) GFP counts.

Figure S6: Gating of flow cytometry raw data of a bacterial sample

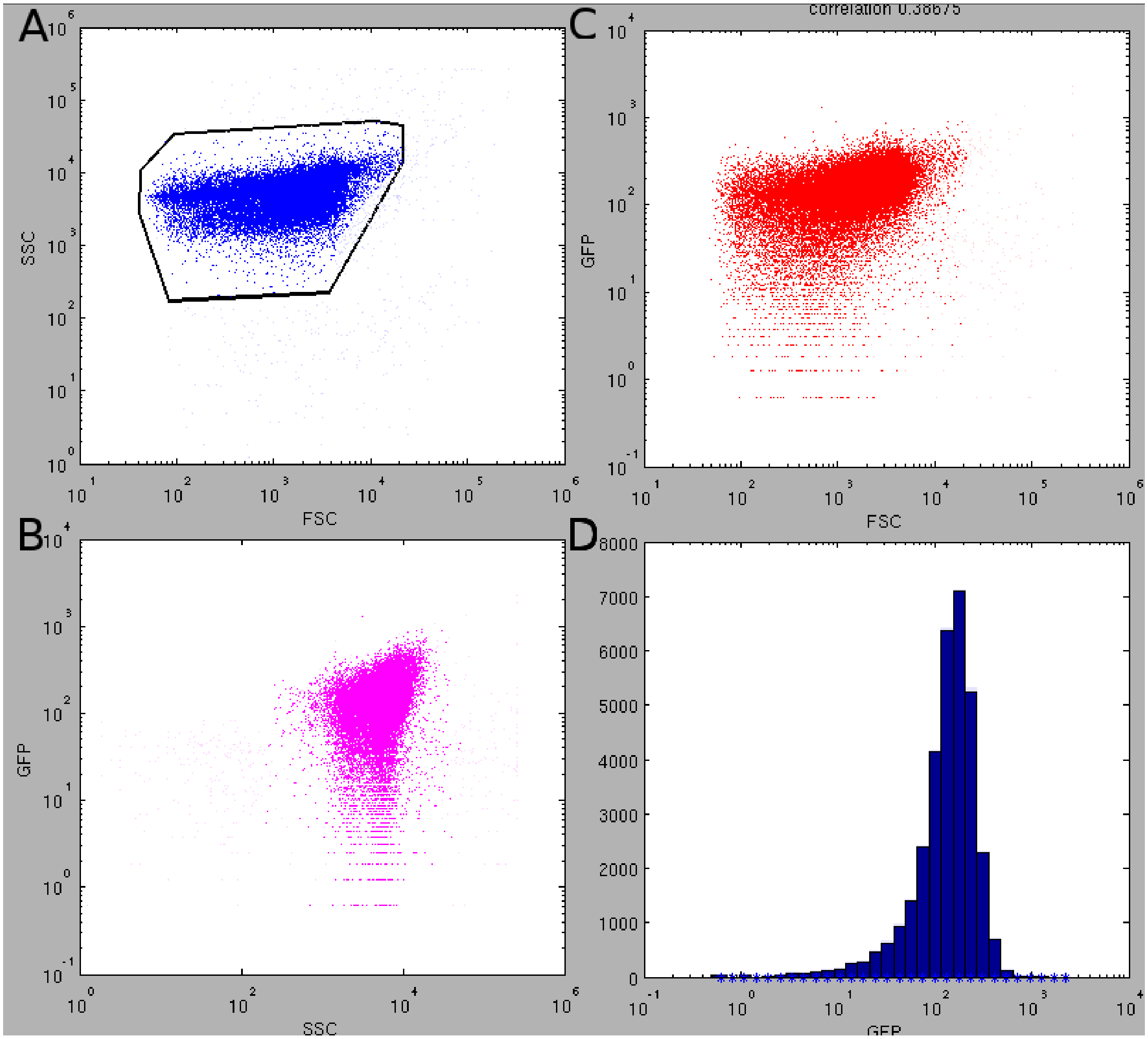

Figure S6. Gating of flow cytometry raw data of a bacterial sample. An example for gated raw data analyzed with LSRII (medium and bacteria). The same gate was used for all samples. (A) SSC against FCS data. The entire bacterial population was gated using FCS and SSC values, and then analyzed for cell number & GFP content. (B) GFP against SSC data. (C) GFP against FSC data. (D) GFP counts.
