# Supplementary material for: Promoter activity dynamics in the lag phase of Escherichia coli
Source: BMC Syst Biol. 2013 Dec 30;7:136. doi: 10.1186/1752-0509-7-136 (PMC3918108; doi:10.1186/1752-0509-7-136)
Supplement: Additional file 3 — Screen plots. 140 promoters normalized screen data plots. [file 1752-0509-7-136-S3.pdf]

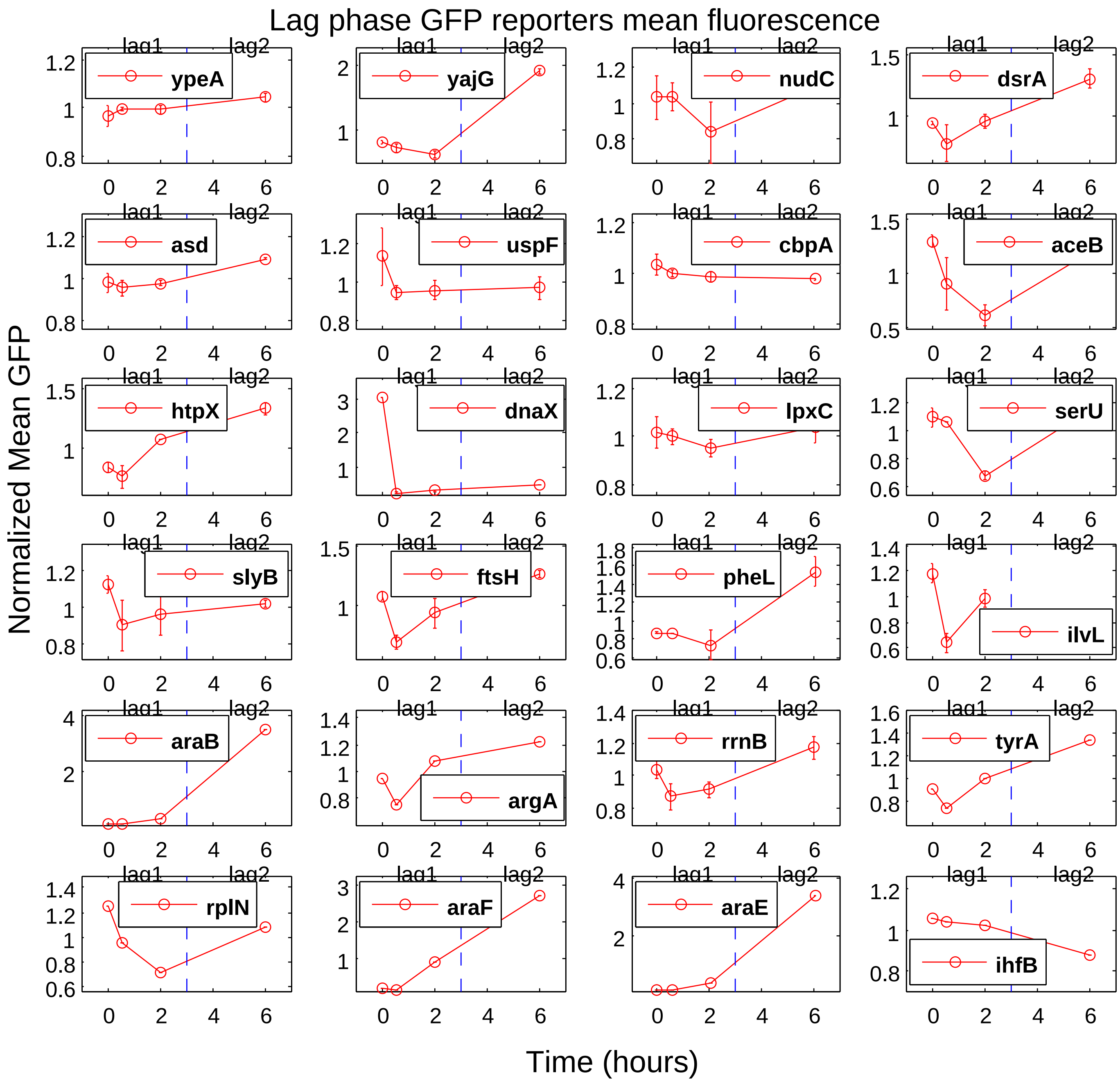
Lag phase GFP reporters mean fluorescence
Normalized Mean GFP
Time (hours)
lag1
lag2
ypeA
yajG
nudC
dsrA
asd
uspF
cbpA
aceB
htpX
dnaX
lpxC
serU
slyB
ftsH
pheL
ilvL
araB
argA
rrnB
tyrA
rplN
araF
araE
ihfB

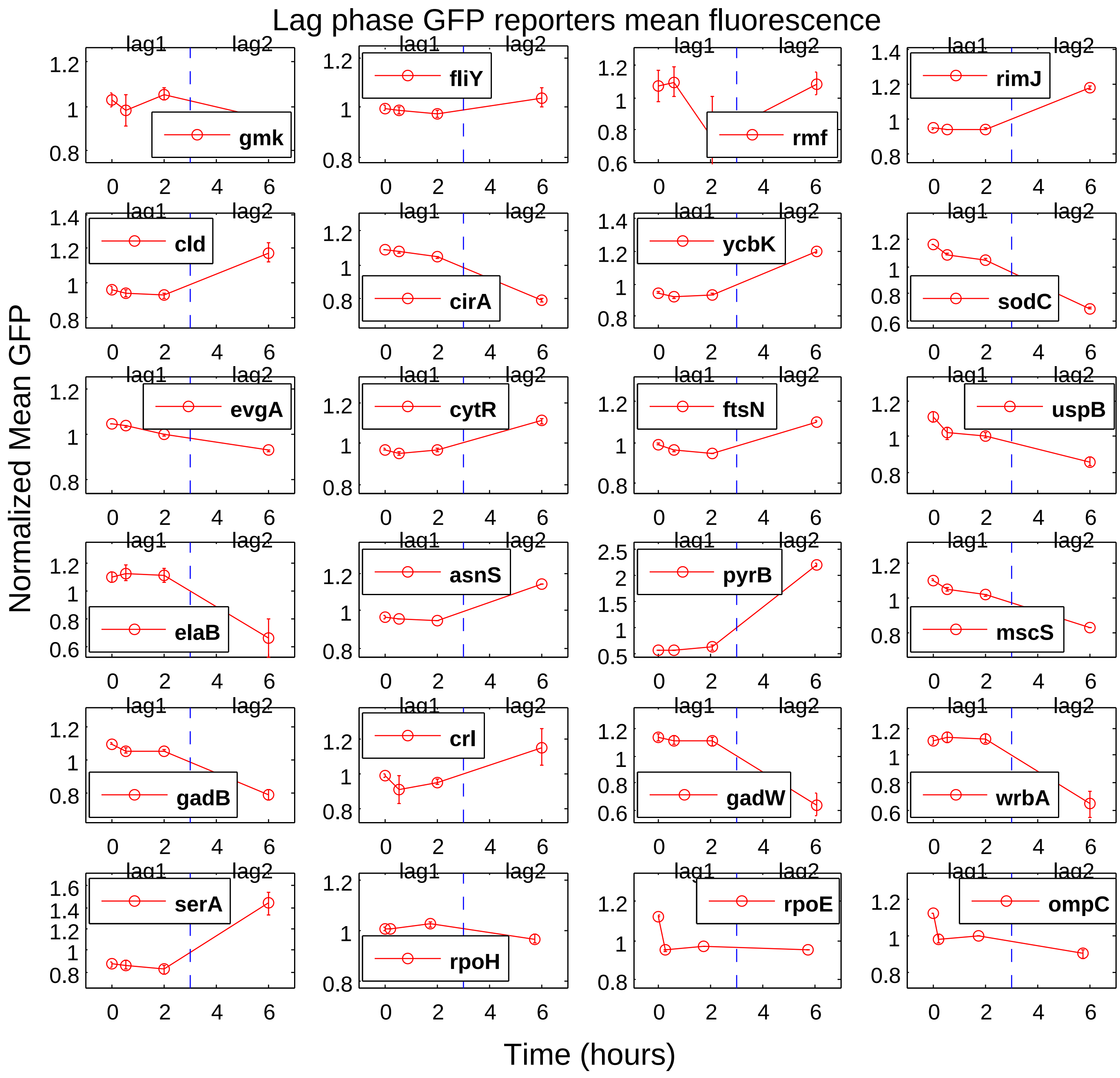
Lag phase GFP reporters mean fluorescence
Normalized Mean GFP
Time (hours)
lag1
lag2
gmk
fliY
rmf
rimJ
cld
cirA
ycbK
sodC
evgA
cytR
ftsN
uspB
elaB
asnS
pyrB
mscS
gadB
crl
gadW
wrbA
serA
rpoH
rpoE
ompC

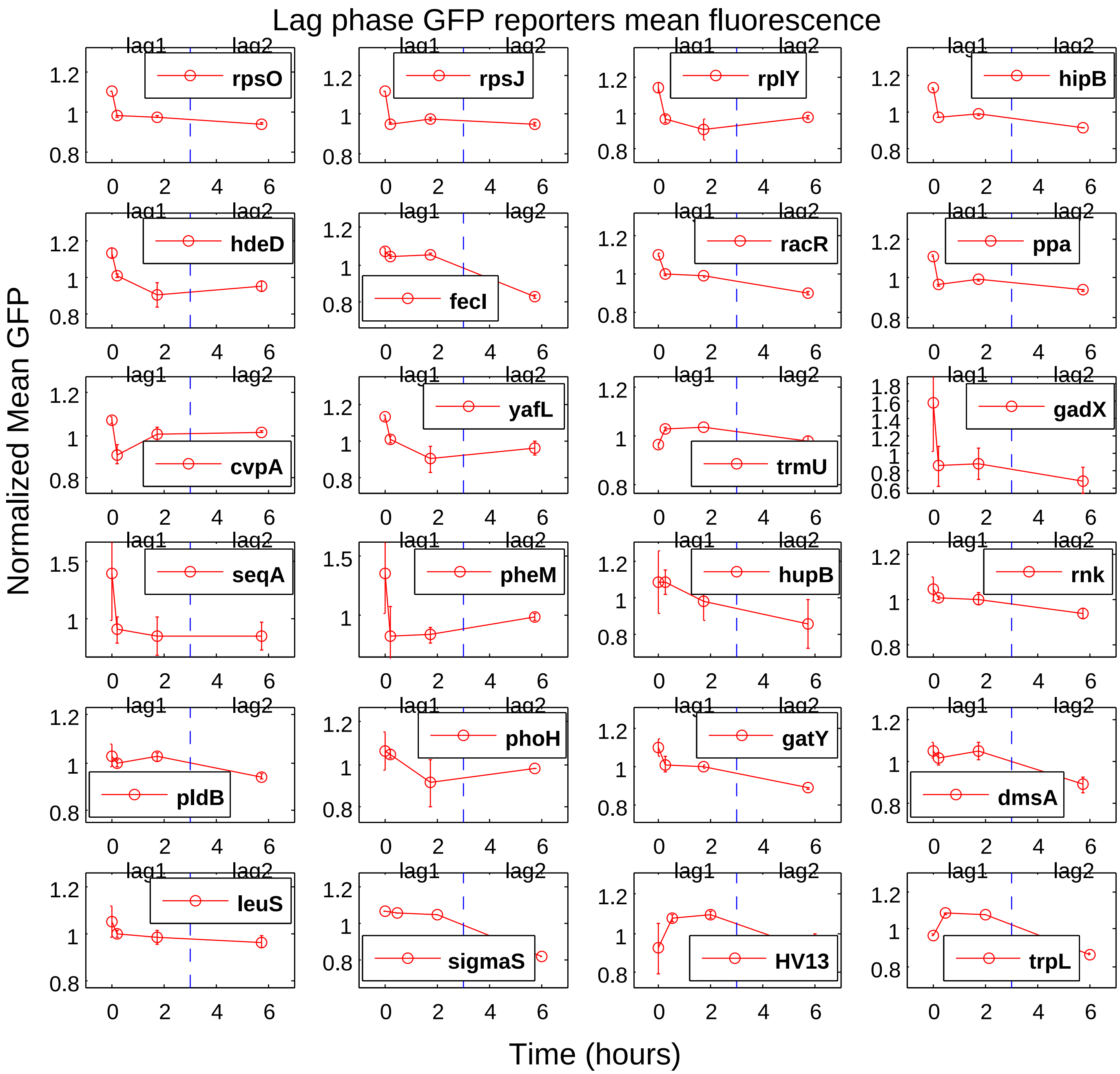
Lag phase GFP reporters mean fluorescence
Normalized Mean GFP
Time (hours)
lag1
lag2
rpsO
rpsJ
rplY
hipB
hdeD
fecI
racR
ppa
cvpA
yafL
trmU
gadX
seqA
pheM
hupB
rnk
pldB
phoH
gatY
dmsA
leuS
sigmaS
HV13
trpL

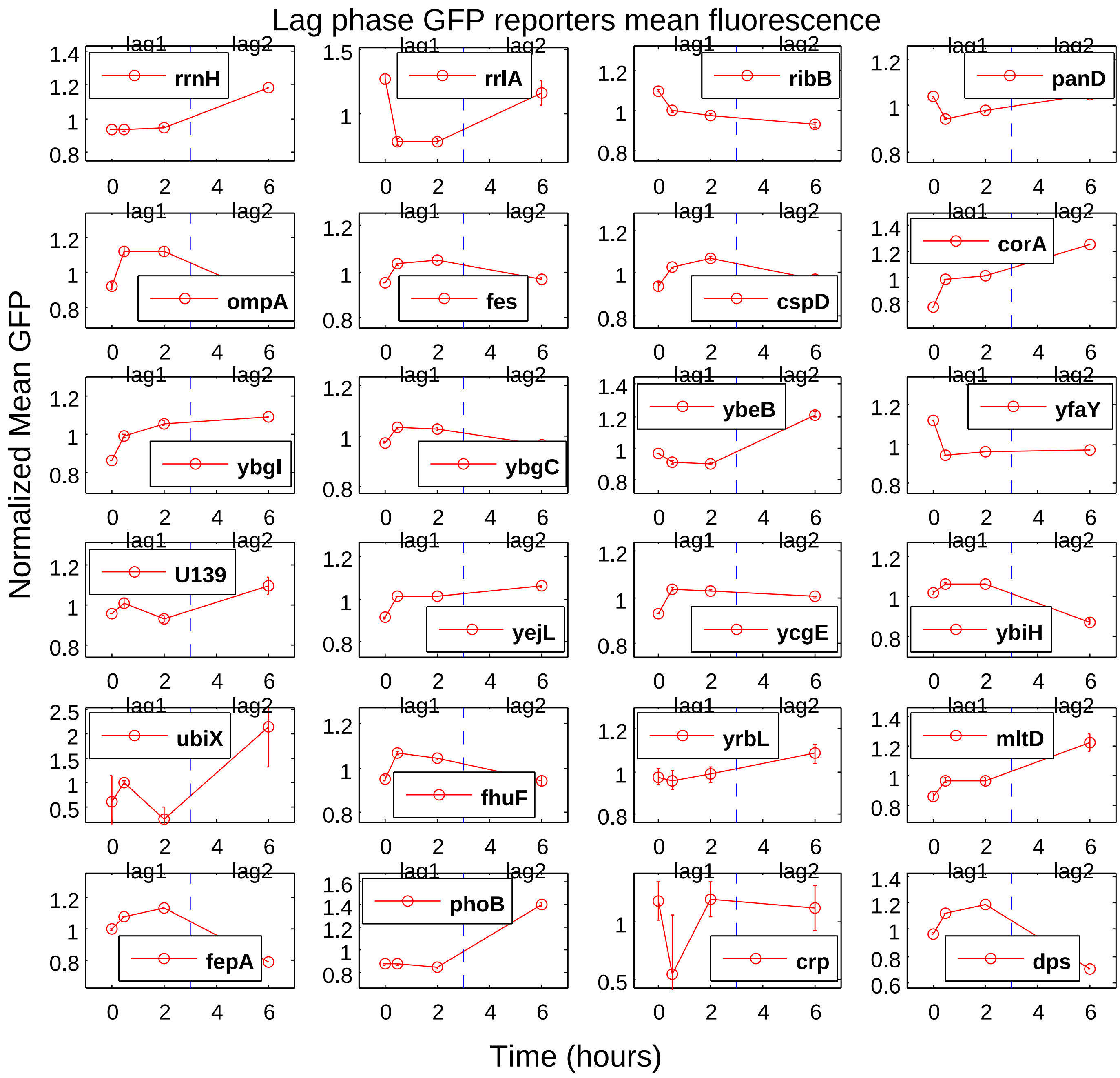
Lag phase GFP reporters mean fluorescence
Normalized Mean GFP
Time (hours)
lag1
lag2
rrnH
rrlA
ribB
panD
ompA
fes
cspD
corA
ybgI
ybgC
ybeB
yfaY
U139
yejL
ycgE
ybiH
ubiX
fhuF
yrbL
mltD
fepA
phoB
crp
dps

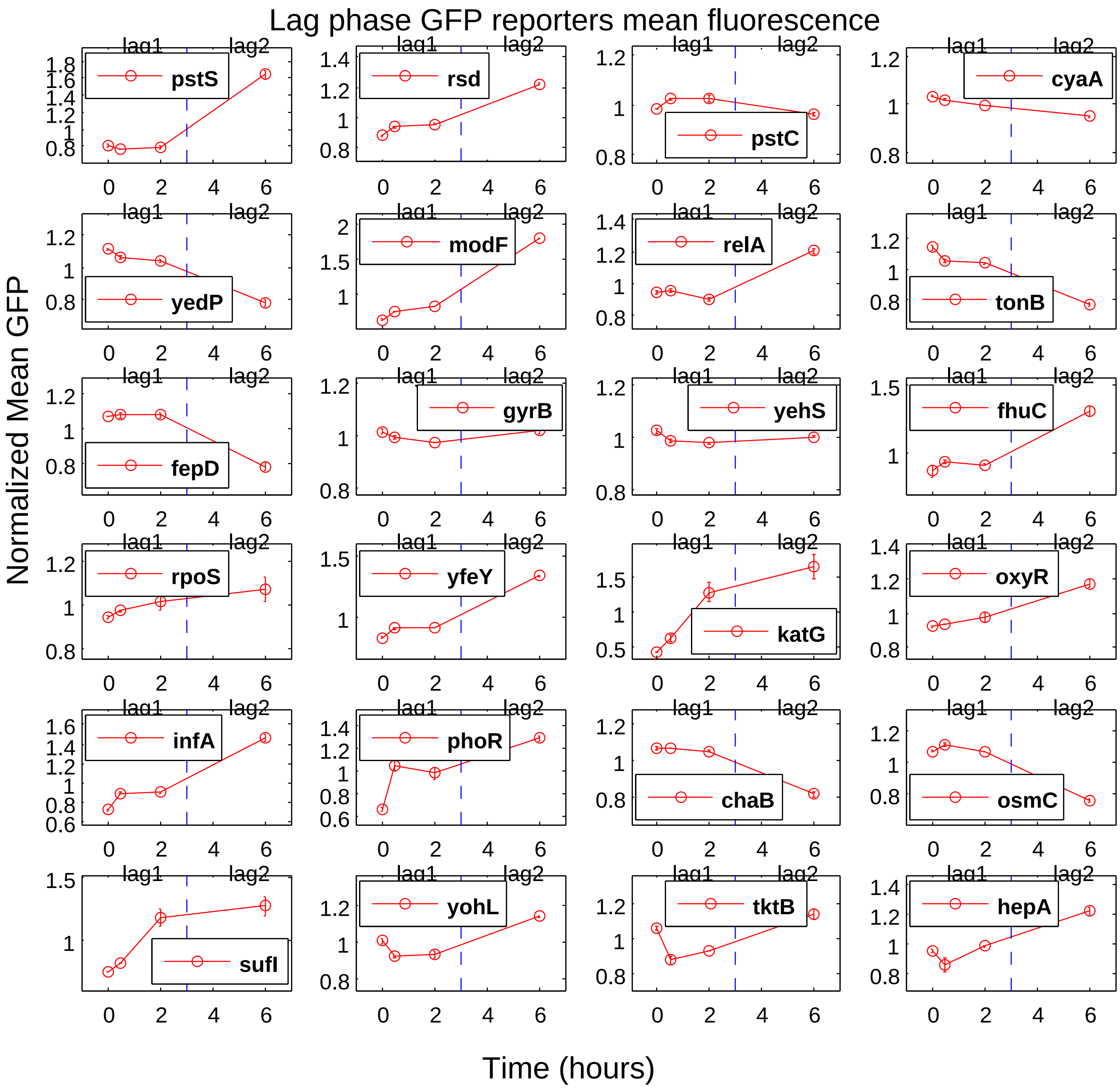
Lag phase GFP reporters mean fluorescence
Normalized Mean GFP
Time (hours)
lag1
lag2
pstS
rsd
pstC
cyaA
yedP
modF
relA
tonB
fepD
gyrB
yehS
fhuC
rpoS
yfeY
katG
oxyR
infA
phoR
chaB
osmC
sufI
yohL
tktB
hepA

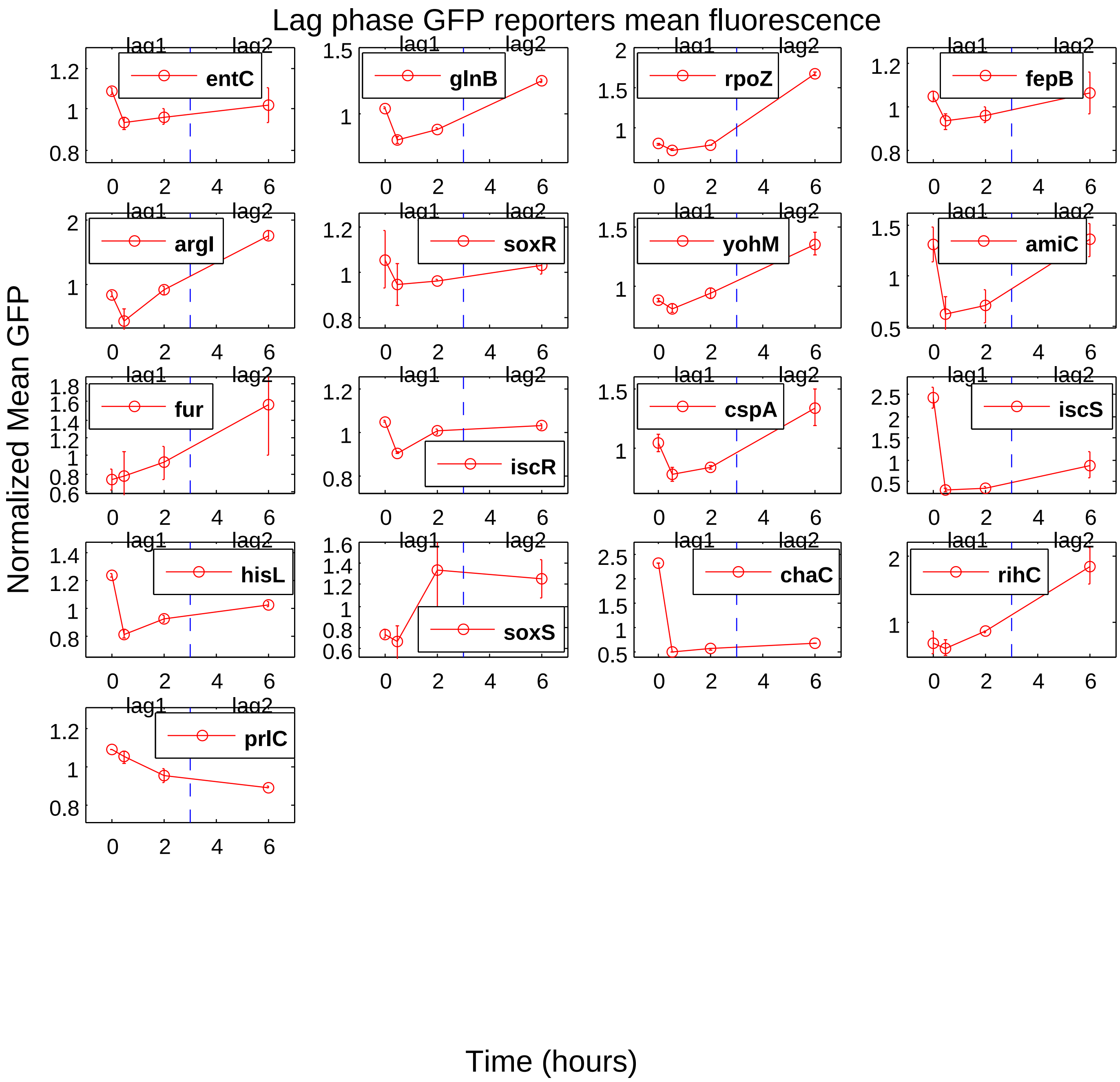

Lag phase GFP reporters mean fluorescence
Normalized Mean GFP
Time (hours)
lag1
lag2
entC
glnB
rpoZ
fepB
argI
soxR
yohM
amiC
fur
iscR
cspA
iscS
hisL
soxS
chaC
rihC
prlC
